# Research Progress on Mammalian Oocyte Vitrification: From Damage Mechanisms to Optimization Strategies

**DOI:** 10.3390/ani16091406

**Published:** 2026-05-03

**Authors:** Kelin Song, Li Wang, Feng Yang, Hongqian Zhu, Qiuyu Meng, Xuelei Han, Ruimin Qiao, Jun Bai, Shuangbao Gun, Tong Yu, Xinjian Li

**Affiliations:** 1 College of Animal Science and Technology, Henan Agricultural University, Zhengzhou 450046, China; 17899312494@163.com (K.S.); 18338155530@163.com (L.W.); chinafy6868@163.com (F.Y.); zhuhongqian@muyuanfoods.com (H.Z.); 13629929273@163.com (Q.M.); hxl014@126.com (X.H.); qrm480@163.com (R.Q.); baijun0320@163.com (J.B.); 2 College of Animal Science and Technology, Gansu Agricultural University, Lanzhou 730070, China; gunsbao056@126.com; 3 Muyuan Foods Co., Ltd., Nanyang 473000, China; 4 Henan Agricultural University Muyuan Livestock Industry Joint Research Institute, Henan Agricultural University, Zhengzhou 450046, China

**Keywords:** mammals, oocytes, vitrification, oxidative stress, molecular mechanisms, regulatory strategies

## Abstract

With the continuous advancement in reproductive biology, oocyte vitrification has become a critical technology for preserving female germplasm and protecting it from environmental disruptions. This technique also eliminates temporal and spatial constraints in animal embryo engineering research. However, during the vitrification of animal oocytes, exposure to low temperatures and high concentrations of cryoprotectants can cause various forms of damage, including cytoskeletal disruption, spindle abnormalities, mitochondrial dysfunction, apoptosis, oxidative stress and epigenetic modifications. These issues are now understood to severely restrict the subsequent developmental competence of oocytes, resulting in lower cleavage and blastocyst formation rates than those of fresh oocytes. Currently, the mechanisms of cryodamage in vitrified oocytes remain poorly understood, and standardized strategies to enhance vitrification efficiency have yet to be firmly established. This review provides a formal overview of the physiological factors underlying oocyte sensitivity to vitrification, alongside the mechanisms of cryodamage and the variables influencing post-thaw survival and reproductive success. It evaluates strategies for mitigating vitrification-induced stress, compares interspecies differences, and addresses current research limitations. By identifying future directions, this review offers new insights for optimizing mammalian oocyte cryopreservation techniques.

## Simple Summary

Oocyte vitrification is an effective method for preserving female germplasm. However, current vitrification procedures for mammalian oocytes can still induce cellular oxidative stress, apoptosis, abnormal spindle distribution, and alterations in epigenetic modifications, thereby affecting subsequent developmental potential. In recent years, numerous studies have focused on incorporating antioxidants and novel cryoprotectants to improve oocyte vitrification protocols and enhance efficiency. Nevertheless, research on the mechanisms underlying cryodamage remains fragmented, and strategies for improving oocyte vitrification outcomes still require further synthesis. This review begins by outlining the fundamentals of oocyte cryo-sensitivity and further summarizes the causes and mechanisms of cryodamage during oocyte vitrification, the factors influencing oocyte survival and reproductive outcomes following vitrification, strategies for regulating vitrification-induced stress in oocytes, a comparison of vitrification processes and outcomes across different species, as well as the limitations of current research and future perspectives.

## 1. Introduction

Advances in animal embryo engineering have established oocyte vitrification as a key strategy for preserving female germplasm and mitigating losses due to major diseases or infections [1]. This technique also overcomes temporal and spatial constraints associated with in vitro embryo culture, embryo transfer, and somatic cell nuclear transfer, thereby facilitating progress in embryonic genetic engineering [2,3]. In the field of animal husbandry, the vitrification of livestock oocytes enables the establishment of germplasm resource banks, thereby accelerating genetic improvement programs in large animals such as pigs, cattle, and sheep [4]. Moreover, it provides technical support for the international exchange of genetic resources while reducing transportation costs by minimizing the need for live-animal shipment [5]. Importantly, long-term preservation of oocytes through vitrification holds significant value for conserving genetic resources of endangered species and maintaining biodiversity [6,7].

Vitrification, first proposed by Rall and Fahy [8] in 1985, was successfully applied to mouse embryo cryopreservation. By exposing cells to high concentrations of cryoprotectants (CPAs) to induce gradual dehydration, then cooling them ultra-rapidly in liquid nitrogen, this technique avoids ice crystal formation by solidifying the solution into a glass-like state at sufficiently high cooling rates [9]. The first successful vitrification of human oocytes resulting in a live birth utilized a solution of 7.1 M ethylene glycol (EG) and 0.6 M sucrose [10]. Following ongoing optimization of vitrification solutions, a protocol combining permeable and non-permeable cryoprotectants (e.g., 15% EG, 15% Dimethyl Sulfoxide (DMSO), and 0.5 M sucrose) has been developed [11,12,13]. Studies have demonstrated that, compared with slow freezing, vitrification of human oocytes can significantly improve the oocyte survival rate (vitrification group 84.7% vs. slow-freezing group 58%) [14]. Owing to its advantage of minimizing ice crystal-induced mechanical damage, vitrification has been extensively applied in the cryopreservation of oocytes from various mammalian species, including economically important livestock such as cattle [15,16], sheep [17], pigs [18,19,20], horses [21], as well as model organisms like mice [22,23,24], and humans [25]. While vitrification is more effective than slow freezing for oocyte preservation [14], the process remains particularly challenging due to the unique structural and physiological characteristics. These challenges arise from the unique physiological characteristics of oocytes, including their large volume, high water content, low membrane permeability, extreme sensitivity of the meiotic spindle to low temperatures, and high intracellular lipid content [2,26,27,28]. These characteristics collectively render oocytes far more vulnerable to cryopreservation-induced damage than other cell types.

In recent years, significant efforts have been undertaken to optimize vitrification protocols for mammalian oocytes to enhance cryopreservation efficiency. However, several challenges in oocyte vitrification remain unresolved [29,30]. On the one hand, the mechanisms underlying cryoinjury have yet to be fully elucidated, particularly those involving oxidative stress, apoptosis, cytoskeletal and spindle disruption, zona pellucida hardening, and epigenetic alterations [30,31,32,33,34,35,36]. On the other hand, effective strategies for mitigating vitrification-induced stress also lack a comprehensive summary. Furthermore, a systematic comparison of vitrification processes and outcomes across species remains lacking. Notably, the cryopreservation efficiency of oocytes from large farm animals, such as pigs, cattle, and sheep, remains relatively low [37]. This review aims to elucidate the mechanisms underlying cryoinjury in mammalian oocytes, identify key factors that affect vitrification success, summarize effective strategies to mitigate vitrification-induced stress, and compare vitrification outcomes across species. The goal is to provide a reference for optimizing vitrification protocols for mammalian oocytes, improving the cryopreservation efficiency of oocytes from large animals, accelerating animal breeding programs, and facilitating the conservation of germplasm resources in endangered species.

## 2. Literature Search and Selection

A systematic literature search was conducted utilizing the PubMed and Web of Science databases for studies published between 1985 and 2026. To ensure a comprehensive retrieval, the following Boolean search string was employed: (vitrification) AND (oocyte OR oocytes) AND (mouse OR mice OR bovine OR cattle OR ovine OR sheep OR porcine OR pig OR human OR humans) AND (antioxidant OR “apoptosis inhibitor” OR “ice recrystallization inhibitor” OR delipidation OR “biosynthetic material” OR “automated device” OR cryoprotectant). The inclusion criteria focused on original research articles investigating mammalian oocyte vitrification, cryoinjury mechanisms, or optimization strategies (e.g., nanomaterials, biosynthetic materials, and antioxidants), provided they reported key outcomes such as survival, cleavage, or blastocyst rates. Conversely, non-English articles, conference abstracts, and duplicates were excluded. The selection process involved an initial independent screening of titles and abstracts by two authors, followed by a rigorous full-text assessment, with any discrepancies resolved through consultation with a third author. From an initial yield of 1058 articles, 178 met the eligibility criteria and were ultimately included. These studies were systematically categorized into themes, including oocyte physiology, cryoinjury mechanisms, efficiency factors, alleviation strategies, and cross-species comparisons.

## 3. Physiological Basis of Oocyte Sensitivity to Vitrification

The sensitivity of mammalian oocytes to vitrification is fundamentally determined by their unique physiological characteristics. First, oocytes possess a large volume relative to their limited plasma membrane surface area, resulting in high intracellular water content and reduced dehydration efficiency during vitrification [26]. Consequently, residual intracellular water may form ice crystals, leading to structural damage [2]. Second, the oocyte exhibits low permeability to cryoprotectants; high concentrations may induce cytotoxicity, whereas insufficient concentrations fail to achieve adequate dehydration, thereby increasing the risk of intracellular ice formation [37]. Third, the meiotic spindle of mature oocytes is acentrosomal and exquisitely sensitive to thermal fluctuations. Even minor cooling triggers microtubule depolymerization, which can result in chromosome misalignment and subsequent aneuploidy [27,38]. Fourth, the high lipid content of oocytes increases their susceptibility to chilling injury, and cryopreservation-induced alterations in lipid droplet morphology can further compromise subsequent developmental competence [28]. Fifth, oocyte vitrification can trigger premature cortical granule exocytosis, resulting in zona pellucida hardening prior to fertilization, thereby impairing sperm penetration even when the oocyte survives [37,39]. Besides, osmotic stress and cryoprotectant toxicity during freezing can disrupt membrane fluidity [26]. Such membrane damage is considered a major factor contributing to the reduced developmental potential of oocytes following cryopreservation [40,41]. Collectively, these physiological characteristics constitute the intrinsic basis for oocyte sensitivity to vitrification. These inherent biological constraints predispose oocytes to cryoinjury, warranting a comprehensive understanding of their sensitivities to systematically elucidate the mechanisms underlying cryoinjury.

## 4. Causes and Mechanisms of Oocyte Vitrification Injury

Oocytes represent the largest category of cells in mammals. Owing to their characteristics of large volume, high water content, and high intracellular lipid content, they are highly susceptible to structural damage during cryopreservation. Vitrification reportedly initiates oxidative stress in oocytes, disrupting intracellular metabolic homeostasis and triggering excessive reactive oxygen species (ROS) production [42]. Excessive ROS directly targets intracellular DNA, proteins, and lipids; among these, lipid peroxidation damages spindle microtubules and microfilaments, leading to spindle depolymerization [43]. Concurrently, oxidative stress impairs mitochondrial function by reducing ATP synthesis, further exacerbating metabolic dysfunction. Under sustained oxidative stress, abnormal structural changes occur in the glycoproteins of the oocyte zona pellucida, resulting in decreased elasticity and zona hardening [44]. Ultimately, the combined effects of oxidative stress, mitochondrial dysfunction, and metabolic disturbances induce abnormalities in epigenetic processes (e.g., DNA methylation and histone modifications) in oocytes, which ultimately lead to embryonic developmental arrest [45].

### 4.1. Oxidative Stress

**Figure 1:**
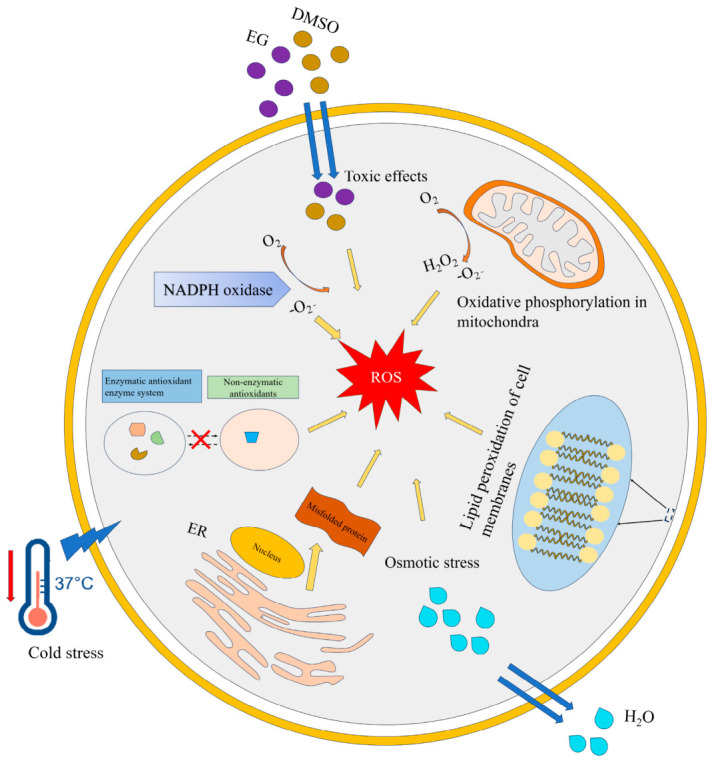
Schematic diagram of ROS generation sources during oocyte vitrification. EG, ethylene glycol; DMSO, dimethyl sulfoxide; ER, endoplasmic reticulum; NADPH, reduced nicotinamide adenine dinucleotide phosphate; O_2_, Oxygen; H_2_O_2_, hydrogen peroxide; -O_2_^−^, superoxide anion; H_2_O, water.

Under physiological conditions, the organism eliminates excessive intracellular ROS via the endogenous antioxidant defense system, thereby maintaining the balance of the intracellular redox status [46]. There are three major types of ROS in cells: hydrogen peroxide (H_2_O_2_), superoxide anion (O_2_^−^) and hydroxyl radical (•OH). ROS production during vitrification and warming of oocytes involves multiple pathways, including cryo-stress, osmotic changes, ice crystal formation, mitochondrial dysfunction, endoplasmic reticulum stress, and imbalances in enzymatic and non-enzymatic antioxidant defense systems [26,33,47] (Figure 1). These factors induce excessive ROS production, which damages mitochondria and reduces ATP synthesis. Numerous studies have demonstrated that vitrification treatment of oocytes increases intracellular H_2_O_2_ levels and decreases glutathione (GSH) levels. Vitrified porcine oocytes showed significantly decreased GSH levels and increased H_2_O_2_ after warming [47]. Vitrification also impaired glucose uptake and decreased GSH and ATP levels, while increasing ROS in mouse oocytes [31]. In a study using the open pulled straw (OPS) device (was a vitrification carrier made in the laboratory) to vitrify porcine metaphase II (MII) oocytes, significant mitochondrial dysfunction was observed, characterized by diminished mitochondrial membrane potential (MMP), decreased ATP production, elevated ROS levels, and dysregulated expression of key apoptosis-related markers, including caspase-3, caspase-8, and caspase-9 [48]. In mammalian oocytes, H_2_O_2_ is generated from superoxide produced by mitochondria and reduced nicotinamide adenine dinucleotide phosphate (NADPH) oxidase [49]. With a relatively long half-life, H_2_O_2_ is further reduced to the more destructive •OH via the Fenton reaction at high concentrations [49], thereby inducing intracellular oxidative stress damage. Overall, vitrification induces overproduction of ROS, leading to lipid peroxidation, DNA damage, and oxidative stress, thereby compromising oocyte survival and developmental competence. Supplementing culture media with antioxidants such as resveratrol, N-acetylcysteine, or ascorbic acid can mitigate oxidative damage. In the future, oxidative stress can be alleviated by supplementing antioxidants or by developing additives that combine antioxidants with other small molecules (e.g., apoptosis inhibitors and regulators of mitochondrial function), thereby achieving efficient oocyte vitrification.

### 4.2. Cytoskeletal and Spindle Disruption

Vitrification can adversely affect the cytoskeleton and spindle structure of oocytes. Guo et al. [50] utilized the OPS device for the vitrification of bovine germinal vesicle (GV) stage oocytes, demonstrating that this procedure can induce varying degrees of cytoskeletal damage. Wei et al. [32] vitrified bovine MII and GV stage oocytes and observed that the expression levels of the cytoskeleton-related genes gap junction protein alpha 1 (GJA1), cytokeratin 8 (CK8), and beta-actin (ACTB) were higher in MII stage oocytes than in GV stage oocytes. Utilizing the cryotop device (Kitazato Corp., Tokyo, Japan), vitrification has been shown to induce structural abnormalities in spindle microtubules of bovine MII oocytes and elevate ROS levels [51]. The oocyte spindle, composed primarily of α and β-tubulin microtubules, is highly sensitive to low temperatures, and its integrity is crucial for proper chromosome segregation during meiosis [52]. Vitrified mouse oocytes reduce microtubule density, disrupting pKIFF11 localization and inhibiting the formation of focused spindle poles via acentriolar microtubule-organizing centers (aMTOCs) [53]. Mammalian somatic cells complete chromosome segregation during mitosis through multiple pathways involving centrosomes [54]. However, oocytes lack canonical centriole structures; the two poles of the spindle are formed via microtubule-organizing centers [55]. Due to the absence of centrosomes, oocytes are highly vulnerable to structural damage and spindle depolymerization during vitrification, caused by extreme osmotic shifts, cryo-injury, and cryoprotectant toxicity [56]. Post-warming, the cell must reassemble the spindle. Without centrioles, oocytes rely on the relocalization of multiple, dispersed MTOCs and associated proteins (e.g., mDia2, pericentrin) to the chromosomal poles, a process that is error-prone and often incomplete. Accordingly, even if morphological recovery occurs, functional defects in the spindle persist, ultimately compromising embryonic developmental potential [57]. Porcine GV stage oocytes subjected to vitrification using the cryolock device (Importadora Mexicana de Materiales para Reproducción Asistida, Sociedad Anónima de Capital Variable, Mexico) exhibited decreased viability (97% in the control group vs. 78% in the vitrification group) and maturation rates (79% in the control group vs. 40% in the vitrification group), along with impaired actin filament organization and chromosomal integrity [30]. Furthermore, research using a mouse model revealed that spindle depolymerization during oocyte vitrification is associated with activation of cathepsin B within lysosomes [58]. These impairments induce abnormal chromosome alignment, along with decreased oocyte survival rate and developmental competence. To address gaps in existing research, future studies could leverage multi-omics approaches to elucidate the regulatory pathways underlying enhanced cytoskeletal stability in MII stage oocytes, thereby improving oocyte vitrification efficiency.

### 4.3. Mitochondrial Dysfunction

As crucial cellular organelles, mitochondria supply energy and maintain intracellular Ca^2+^ homeostasis [59]. Under physiological conditions, mitochondrial Ca^2+^ homeostasis, ATP synthesis, and ROS production are tightly regulated, maintaining a delicate balance [60]. Mitochondria are composed of inner and outer membranes, with continuous electron transfer across the inner membrane [61]. Electrons are transported through respiratory chain complexes I-IV and ultimately combine with oxygen [62]. This process establishes an electrochemical gradient across the membrane, known as the MMP, that drives oxidative phosphorylation, converting ADP to ATP [63]. However, compared to mammalian somatic cells, oocytes exhibit lower basal ATP levels. During vitrification and warming, extreme temperature fluctuations severely impair mitochondrial function. Vitrified mouse MII oocytes showed decreased mitochondrial copy numbers, altered distribution, and reduced ATP production [64,65]. Similar cytoskeletal damage has also been reported in other species, such as pigs and cattle [66,67]. Vitrification-induced oxidative stress impairs respiratory chain function, creating a vicious cycle of ROS accumulation that leads to critical molecular degradation, lipid peroxidation, and ultimately, cell death [33]. Furthermore, studies indicate that vitrification alters mitochondrial thermostasis and impairs mitochondrial quality in mouse oocytes [12]. These studies collectively demonstrate that vitrification impairs mitochondrial function through oxidative stress, altered MMP, and reduced ATP production. Future research may improve vitrification efficiency by deciphering temperature-mediated mitochondrial damage and stage-specific mitochondrial tolerance.

### 4.4. Damage to the Zona Pellucida

The zona pellucida (ZP) is now understood to play a critical role in early oocyte development [68]. Mouse oocytes with an intact ZP exhibit significantly higher post-vitrification survival rates compared to ZP-deficient oocytes, which is closely associated with their enhanced mechanical stability [69], highlighting the structural importance of the ZP during vitrification. Ultrastructural analysis of human oocytes following cryopreservation revealed a significant attenuation in cortical granule density [70]. Cryoprotectants and freezing–thawing processes can induce elevated intracellular Ca^2+^ levels, leading to premature cortical granule exocytosis and subsequent ZP hardening [34]. Premature release of cortical granules causes hardening of the ZP in oocytes, leading to a significant reduction in fertilization rate after cryopreservation. Studies in human oocytes have demonstrated abnormal reductions in cortical granule number and density using a cryotip device (Paillettes Crystal 133 mm; CryoBioSystem, Paris, France), potentially contributing to ZP hardening [39,71]. These findings collectively indicate that vitrification-induced ZP damage can impair sperm–oocyte interaction, reduce fertilization rates, and decrease early embryonic development. Therefore, in human reproductive medicine, intracytoplasmic sperm injection (ICSI) is commonly used to fertilize vitrified oocytes, thereby effectively circumventing the decline in fertilization rate caused by zona pellucida hardening. Similarly, this methodology is applicable to the vitrification of oocytes from endangered species, where it may mitigate the attenuation of developmental rates associated with zona pellucida hardening.

### 4.5. Induction of Apoptosis

Mammalian oocyte apoptosis occurs primarily through the mitochondria-mediated intrinsic pathway and the death receptor-mediated extrinsic pathway [72]. Current evidence suggests that granulosa cell apoptosis disrupts the supply of critical signaling molecules and small metabolites required for oocyte meiotic progression, thereby increasing the susceptibility of mammalian oocytes to apoptosis [73,74]. Meanwhile, oocytes at different developmental stages exhibit varying sensitivities to the activation of apoptotic pathways. Studies have demonstrated that, compared with mature rat oocytes, immature oocytes are more susceptible to H_2_O_2_-induced apoptosis, accompanied by phenotypic changes, including membrane degeneration and cell shrinkage [75]. In addition, elevated Ca^2+^ levels inside and outside the cell are major contributors to oocyte apoptosis. Sustained high intracellular Ca^2+^ concentrations induce oocyte apoptosis by increasing intracellular ROS levels or triggering FAS receptors [76,77]. Gao et al. [35] reported that supplementing vitrification/warming solutions with 3-methyladenine in mouse immature oocytes led to significantly higher mRNA and protein levels of beclin-1 (an autophagy marker) and caspase-3 compared to fresh oocytes. The surge in calcium ions within oocytes during vitrification elevates hydrogen peroxide levels. The persistent accumulation of ROS upregulates the Bax/Bcl-2 expression ratio, alters MMP, triggers cytochrome release, and ultimately activates the caspase cascade [78,79]. In summary, vitrification induces apoptosis via pathways including Fas/FasL, TNFR/TNF-α, and ROCK. Upregulation of apoptosis-related genes may serve as an early indicator of reduced developmental potential in vitrified oocytes. To mitigate apoptosis during oocyte vitrification, future research could focus on developing non-toxic cryoprotectants and targeted apoptosis inhibitors to suppress ROS accumulation and the subsequent activation of apoptotic signaling pathways.

### 4.6. Alterations in Epigenetic Modifications

Accumulating evidence indicates that oocyte vitrification is associated with widespread epigenetic perturbations; however, the extent to which these alterations are functionally relevant remains incompletely understood. Several studies have reported disrupted DNA methylation patterns following vitrification, accompanied by the downregulation of imprinted genes such as insulin-like growth factor 2 receptor (*IGF2R*), protein phosphatase 1 regulatory subunit 9A (*PPP1R9A*), and paternally expressed 3 (*PEG3*) [80]. In parallel, alterations in histone modifications have been consistently observed across species. Furthermore, Spinaci et al. [36] observed that vitrification of porcine oocytes induced alterations in site-specific histone acetylation, notably at histone H3 lysine 9 (H3K9ac), H4 lysine 5 (H4K5ac), and H4 lysine 21 (H4K21ac), as well as a reduction in H3 lysine 9 trimethylation (H3K9me3). Recent studies further suggest that vitrification may disrupt histone lactylation, as evidenced by the downregulated expression of lactate dehydrogenase A (LDHA), lactate dehydrogenase B (LDHB), and E1A binding protein p300 (EP300) in vitrified mouse oocytes [81]. Meanwhile, Vitrification reduced Sirtuin (*SIRT1*) expression, leading to aberrant H3K9 acetylation, DNA methylation, and expression of imprinted genes such as gene trap locus 2 (*Gtl2*) and *Peg3* [82]. Although vitrification reportedly disrupts epigenetic regulation, including DNA methylation and histone modifications, direct comparisons remain challenging due to variations in experimental design, species, and analytical techniques. In addition, vitrification can cause vacuolization [83], increase mitochondria-smooth endoplasmic reticulum (M-SER) aggregates and mitochondria-vesicle complexes [39] and alter Ca^2+^ distribution, ultimately leading to morphological abnormalities and reduced developmental competence.

## 5. Key Factors Affecting Post-Vitrification Survival and Reproductive Success

The success of mammalian oocyte vitrification is determined by a complex interplay of multiple variables rather than any individual factor in isolation. These factors include the developmental stage of oocytes [32], the type and concentration of cryoprotective agents [84,85], vitrification devices and operational conditions (such as equilibration temperature and duration) [86], intracellular lipid content [87], cooling medium temperature [50,88], sample volume [89], physicochemical properties and osmolarity of vitrification solutions, as well as operator dependent technical variability. Therefore, accurate identification and optimization of the key limiting factors are essential for improving vitrification outcomes in mammalian oocytes.

### 5.1. Oocyte Developmental Stage

Among the factors limiting mammalian oocyte vitrification efficiency, the developmental stage of the oocyte is a key determinant. For farm animals, such as pigs, cattle, and sheep, understanding how oocytes respond to vitrification across developmental stages is crucial for optimizing protocol design and improving survival rates. From a morphological perspective of mammalian oocytes, the ooplasm diameter of immature oocytes is smaller than that of mature oocytes, and immature oocytes have lower developmental competence [90]. In vitrified mouse GV and MII oocytes using the cryolock device (Cat. No. CL-R-CT, Biotech, Inc., California, USA), the incidence of abnormal meiotic spindles was higher in MII oocytes [91]. MII oocytes contain cold-sensitive meiotic spindles, making them more susceptible to low-temperature damage during vitrification, whereas GV oocytes can avoid spindle damage [92,93]. Conversely, when bovine GV and MII-stage oocytes were vitrified, GV-stage oocytes exhibited a lower survival rate (25.90%) compared with MII-stage oocytes (35.60%) [32]. These findings suggest significant differences in cryopreservation efficiency between GV and MII oocytes across mammalian species. This may be attributed to the distinct morphological and structural features of mammalian oocytes at different developmental stages. For instance, compared with mature mammalian oocytes, immature oocytes exhibit characteristics such as reduced microtubule post-translational modifications, lower adenosine triphosphate (ATP) levels, decreased amounts of maturation-promoting factor, impaired glutathione synthesis capacity, and sluggish energy metabolism [94,95], which lead to marked differences in their post-vitrification performance. Therefore, appropriately supplementing exogenous substances according to the structural characteristics of different developmental stages of mammalian oocytes can significantly improve the efficiency of oocyte vitrification.

### 5.2. Types of Cryoprotectants

CPAs are commonly classified into permeating and non-permeating agents. Permeating CPAs, such as EG and DMSO, inhibit ice crystal formation by disrupting hydrogen bonding between water molecules [96]. Brewer et al. [97] demonstrated that a combination of EG and propylene glycol (PROH) for cryopreserving MII oocytes yielded higher MMP and lower ROS compared to an EG and DMSO regimen. However, the cytotoxic effects of permeating CPAs remain a significant concern in cell freezing. Studies have shown that even low concentrations of DMSO (around 0.1%), particularly in vitro, may induce significant alterations in transcriptomic, proteomic, and epigenetic profiles in human somatic cells [98]. Within the normal concentration range for oocyte vitrification (not exceeding 15%), the inclusion of DMSO exerts highly detrimental effects on oocytes [84]. These findings collectively indicate that the toxic effects of permeable CPAs (DMSO and EG) can impair subsequent oocyte development. Consequently, there is an urgent need to identify non-permeable cryoprotectants that can either fully replace or partially reduce the proportion of permeable CPAs in vitrification protocols.

Non-permeating CPAs, due to their high molecular weight, cannot enter cells easily. Instead, they increase solution viscosity, promote rapid cellular dehydration, and reduce the rate of ice crystal formation. Common non-permeating cryoprotectants include sucrose, ficoll, trehalose, polyvinylpyrrolidone, and antifreeze proteins (AFPs). Such cryoprotectants primarily promote cellular dehydration by increasing extracellular osmotic pressure. Among these, sucrose and trehalose are commonly used [99] and can maintain the balance between the rapid dehydration process prior to cell freezing and the rehydration process after thawing. Supplementing trehalose during vitrification could enhance the membrane stability of ovine oocytes during both cryopreservation and subsequent heat exposure [85]. This effect may be attributed to the ability of sucrose and trehalose to alter the osmotic pressure of the extracellular fluid of oocytes and reduce osmotic damage to oocytes [100]. In summary, during oocyte vitrification, selecting the optimal combination of CPAs based on species-specific characteristics is crucial for improving vitrification efficiency.

### 5.3. Vitrification Devices and Equilibration Temperature and Times

**Figure 2:**
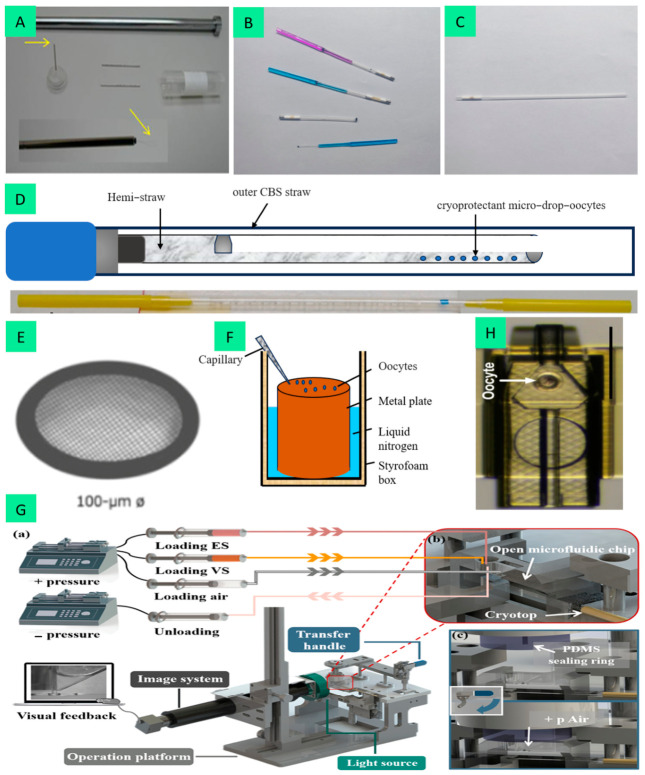
Representative images of different oocyte vitrification devices: (**A**) cryoloop, reproduced from [101]; (**B**) cryotop; (**C**) open pulled straw (OPS); (**D**) hemi-straw, adapted from [102]; (**E**) electron microscopy grids, adapted from [103]; (**F**) solid surface vitrification (SSV); (**G**) microfluidic vitrification device, reproduced from [105]: (**a**) Schematic representation of the vitrification system configuration; (**b**) The microfluidic chip and Cryotop are fixed on the operation stage; (**c**) Schematic diagram of the transfer of oocytes to the Cryotop; (**H**) 3D photopolymerized device, reproduced from [106]. All reproduced/adapted images are distributed under the Creative Commons Attribution 4.0 International License (CC BY 4.0).

It is well-established that the selection of vitrification devices during the process determines the cooling and warming rates, which are among the main factors limiting vitrification efficiency. Common vitrification devices include the cryoloop [101], OPS, cryotop, hemi-straw [102], electron microscopy grids [103], solid surface vitrification (SSV) [104], Microfluidic vitrification device [105] and 3D photopolymerized device [106]. Representative images of each device are shown in Figure 2. These devices employ different materials and offer distinct advantages (Table 1). Notably, Yagoub et al. developed a 3D photopolymerization-based device in 2022 capable of achieving a 1000-fold reduction in CPA volume while streamlining the procedural workflow for mouse oocyte vitrification [106]. In addition, researchers have developed automated vitrification devices that enable the automated vitrification of mouse oocytes through linear CPA loading and precise regulation of cryoprotective solution volume, and results showed that oocytes vitrified with this automated device had higher survival (80.44% vs. 73.35%), cleavage (54.17% vs. 43.73%), and blastocyst rates (32.95% vs. 23.67%) compared with the manual cryotop device [107]. In research on mammalian oocyte vitrification, ongoing efforts have aimed to reduce the volume of cryoprotectants to improve vitrification efficiency. There are significant differences in the cooling rates of different vitrification carriers. Simulation experiments have demonstrated that cryoloop has the highest efficiency, reaching up to 100,000 °C/min [108], while the cooling rate of cryotop ranges from 37,500 °C/min [109] to 40,000 °C/min [86]. Although cryoloop offers superior cooling rates, practical applications have demonstrated that cryotop is more suitable for sheep oocytes, with a significantly higher survival rate (83.84%) compared to the traditional straw group (63.43%) [110]. Notably, a higher cooling rate during oocyte vitrification is associated with better vitrification outcomes. This finding accounts for the significant differences in the survival rates of cold-sensitive oocytes observed with different vitrification carriers.

Beyond the choice of vitrification carrier, exposure conditions to CPAs also significantly influence the functionality and subsequent development of vitrified oocytes. Among these conditions, exposure temperature and duration are critical, and interspecies differences are particularly pronounced. Prolonging equilibration to 10 min reduced the survival and blastocyst rates of mouse oocytes [111]. Exposing bovine oocytes to equilibration solution for 150 s at 38.5 °C improved oocyte quality and blastocyst rates after vitrification/warming [112]. Before vitrifying goat oocytes with the cryotop device, exposure to 10% DMSO or 10% EG for 1 or 3 min resulted in higher viability and developmental rates [113]. Similarly, vitrified/warmed sheep MII oocytes using a 0.25 mL straw (angled-cut straw tip used as a vitrification carrier) showed no significant difference in survival rates after 5, 7, or 10 min of equilibration [114]. This phenomenon may be attributed to increased oocyte permeability and CPA toxicity at higher temperatures [115,116]. The above studies overlap in their assertion that the efficiency of vitrification is strongly influenced by the equilibration time in the solution and that the vitrification solution should be selected based on the physiological characteristics of mammalian cells to achieve better results.

### 5.4. Lipid Content

Higher intracellular lipid levels are associated with lower cryotolerance [117]. Under low-temperature conditions, membrane lipids undergo a phase transition from the liquid-crystalline to the gel state [118]. This phase change directly disrupts membrane architecture, thereby interfering with functional contacts among lipid droplets, the endoplasmic reticulum, and mitochondria, ultimately compromising cellular metabolic energy homeostasis [119]. Moreover, the morphology of lipid droplets influences cold sensitivity. In bovine GV-stage oocytes, lipid droplets are structurally simple, appearing as dense and homogeneous spheroids that remain morphologically stable at low temperatures and help maintain membrane integrity, whereas porcine GV-stage oocytes contain structurally complex lipid droplets, including homogeneous dark droplets and grey droplets with electron-lucent striations involved in lipid metabolism, which makes them more susceptible to phase transition and peroxidation during cryopreservation, thereby increasing cryo-damage [120]. To reduce lipid content in oocytes, researchers have developed several strategies, including centrifugation, mechanical delipidation via micromanipulation, and chemical delipidation. A study revealed that mechanical delipidation of porcine oocytes significantly reduced mitochondrial distribution, decreased ROS levels and cleavage rates (delipidated group 21.4% vs. non-delipidated group 10.4%) after vitrification [121]. In addition to the aforementioned factors, selecting an appropriate cooling medium temperature is critical for improving the efficiency of mammalian oocyte vitrification. Liquid helium (LHe), which has a substantially lower temperature than liquid nitrogen (LN_2_), has been reported to yield superior cryopreservation outcomes in immature bovine germinal vesicle (GV) oocytes compared with LN_2_-based vitrification [50,88]. The volume of the vitrification droplet is another important determinant of vitrification efficiency. In conventional practice, the droplet volume is typically restricted to less than 1 μL to facilitate ultra-rapid cooling and to minimize ice crystal formation [89]. Similarly, the osmolarity of vitrification solutions plays a pivotal role. During the vitrification process, oocytes are exposed to dramatic osmotic changes, transitioning from approximately 280 mOsm (culture medium) to around 2700 mOsm (equilibration solution), and subsequently to about 5600 mOsm in the vitrification solution. The warming process involves a reverse osmotic shift [26]. These abrupt osmotic fluctuations can impose severe stress on the cell membrane, potentially leading to significant cellular damage.

**Table 1:** Advantages of different vitrification devices.

Devices	Steps	Advantages	Reference
Cryoloop	Oocytes are transferred onto a thin film supported by a nylon loop, followed by storage in LN_2_.	This approach requires a minimal volume of cryoprotectant and facilitates rapid cooling.	[122]
open pulled straw (OPS)	Straws are heated-pulled to create a narrow tip, onto which multiple oocytes are loaded before storage in LN_2_.	Increases cooling rate and reduces cellular damage.	[123]
Cryotop	Under a stereomicroscope, oocytes are loaded onto a plastic strip tip, submerged in LN_2_, and stored long-term.	Enables ultra-rapid cooling and warming rates.	[86]
Hemi-straw	A 0.25 mL straw is cut into a 1 cm × 0.5 mm strip, onto which oocytes are aligned prior to storage in LN_2_.	Simple operation with a large contact surface area with LN_2_.	[86]
solid surface vitrification (SSV)	A solid medium is pre-cooled in LN_2_, onto which a droplet of CPA–oocyte mixture is deposited for long-term storage in LN_2_.	Easy to perform and cost-effective.	[124]
Electron microscopy grids	Oocytes are loaded onto electron microscopy grids, blotted to remove excess CPAs, and directly plunged into LN_2_ for storage.	High-throughput processing capability.	[125]
Automated vitrification	Precise regulation of CPA concentration around oocytes can be achieved through linear loading and removal protocols.	Cryoprotectant volume to automate freezing.	[107]
3D photopolymerized device	Place single cells/embryos in pods, place multiple pods in a Garage, repeat vitrification heating and cycling.	Minimal volume of cryoprotectant required.	[106]

## 6. Strategies to Improve Oocyte Vitrification Efficiency

Improving the quality of vitrified oocytes is key to advancing the rapid advancement in assisted reproductive technology (ART) and the efficient in vitro production of embryos. Currently, the main strategies to enhance vitrification efficiency include reducing oxidative stress, inhibiting ice crystal formation, suppressing apoptosis, delipidation or lipid content reduction, and stabilizing the cytoskeleton, as well as the application of novel biosynthetic materials and the development and application of novel vitrification devices.

### 6.1. Reducing Oxidative Stress

During vitrification, excessive ROS generation induces oxidative stress. Numerous studies have reported that supplementing antioxidants such as ascorbic acid, melatonin, L-carnitine, mitoquinone (MtQ), chlorogenic acid, resveratrol, astaxanthin and spermidine can mitigate cryo-induced damage. L-carnitine is a class of small-molecule substances that can promote fatty acid β-oxidation and ATP synthesis. Fatty acids in the cytoplasm cannot directly enter mitochondria for oxidative decomposition and must rely on carnitine, a small-molecule carrier, to translocate into mitochondria [126,127]. MtQ is an antioxidant that can deliver ubiquinone into mitochondria. It alleviates cellular oxidative stress by reducing lipid peroxidation, prevents apoptosis induced by leakage of superoxide radicals from the mitochondrial respiratory chain, and increases intracellular glutathione levels [128,129]. Thus, mtQ acts as an antioxidant during vitrification and plays a critical role in mitigating oxidative damage in cells. Resveratrol and astaxanthin are both common antioxidants; resveratrol can act on multiple molecular targets simultaneously to effectively reduce ROS production in cells, while astaxanthin scavenges ROS and oxygen free radicals and inhibits the process of lipid peroxidation [130,131,132]. The applications of the above-discussed antioxidants in mammalian oocyte vitrification are summarized in Table 2.

### 6.2. Inhibiting Ice Crystal Formation

AFPs, originally identified in deep-sea fish and subsequently isolated from fungi and plants, have been extensively studied for their ability to modify ice crystal structure and inhibit ice formation [133,134]. AFPs have been successfully used for the cryopreservation of oocytes and embryos across different species. For example, Lee et al. [135] demonstrated that supplementing the vitrification solution with 0.05–0.1 mg/mL of various antifreeze proteins, namely, Flavobacterium frigoris ice-binding proteins (FfIBP), Glaciozyma sp. ice-binding proteins (LeIBP), and AFP-III from bacteria, yeast, and fish during mouse oocyte cryopreservation significantly improved oocyte survival (85.0% vs. 75.0%), cleavage (81.2% vs. 58.7%), and blastocyst formation rates (76.8% vs. 58.7%). Li et al. [136] compared the effects of the straw vitrification device and programmed freezer on the vitrification of ovine oocytes and found that supplementation of 10 μg/mL antifreeze proteins from *Anatolia polita* (ApAFP914) into the vitrification solution could significantly improve the survival rate of in vitro fertilized ovine embryos (97.17% in the vitrification group vs. 72.47% in the slow freezing group). Leal et al. [137] vitrified feline oocytes, and supplementation with 0.1–1 μg/mL AFP-I increased the cooling rate to 1700 °C/min; compared with the control group, this treatment improved the survival rate of feline oocytes (75% in the control group vs. 89–90% in the AFP I-supplemented groups), enhanced mitochondrial activity, and reduced intracellular ROS levels. However, high production costs limit their widespread use. Notably, synthetic ice crystal inhibitors have also come into view. For instance, Santos et al. [138] found that the synthetic ice blockers Supercool X-1000 (SC) and carboxylated ε-poly-L-lysine (COOH-PLL) exerted beneficial effects in the vitrification of porcine oocytes.

### 6.3. Suppressing Apoptosis

Apoptosis induced during vitrification can be mitigated using apoptosis inhibitors, thereby improving oocyte survival and developmental competence. Treatment with the pan-caspase inhibitor benzyloxycarbonyl-Val-Ala-Asp -fluoromethyl ketone (Z-VAD-FMK) reduced DNA damage and caspase activity in vitrified feline oocytes, yielding a maturation rate (53.13%) comparable to fresh controls (65.38%) and similar cleavage rates (34.38% vs. 31.78%) [139]. These findings substantiate that excessive caspase activation is a key mediator of cryo-induced injury. However, it should be noted that caspase inhibition primarily targets downstream apoptotic execution and may not fully prevent upstream cellular stress triggered by vitrification. Pre-treatment of bovine oocytes with a 10 μM Rho-associated coiled-coil kinase (ROCK) inhibitor (R)-(+)-trans-N-(4-pyridyl)-4-(1-aminoethyl)cyclohexanecarboxamide (Y-27632) for 2 h significantly improved survival, cleavage, and blastocyst rates after vitrification [140]. Thus, adding apoptosis inhibitors during vitrification and warming reduces activation of mitochondrial and extrinsic apoptotic pathways, minimizes cellular damage, and enhances oocyte cryosurvival and developmental potential.

### 6.4. Delipidation or Lipid Reduction

Given the high lipid content and extreme cold sensitivity of oocytes, reducing cytoplasmic lipid levels is an effective strategy to improve cryotolerance. Although mechanical delipidation via microinjection through the zona pellucida effectively reduces lipid content, the procedure poses a significant risk to cellular structural integrity [141]. Alternatively, adding lipolytic agents to oocyte culture media can safely reduce lipid content and improve freezing tolerance. Fu et al. [142] demonstrated that supplementing maturation media with 10 μM forskolin significantly decreased lipid levels and increased post-vitrification survival. During the vitrification of feline oocytes, supplementation with 2 mg/mL levocarnitine can significantly improve the survival rate and ATP levels of vitrified oocytes and reduce intracellular ROS levels [143]. During the vitrification of bovine oocytes using the OPS device, supplementing with 1 μM β-nicotinamide mononucleotide (NMN), 2.5 μM berberine (BER), or 1 μM cordycepin reduced lipid content, downregulated lipid synthesis-related genes, and decreased ROS and apoptosis levels [15]. During the vitrification of feline oocytes, reducing lipid content using lipid modulators such as conjugated linoleic acid, forskolin, and L-carnitine improved oocyte viability (74% in the experimental group vs. 53% in the control group) [144]. Forskolin acts as a lipolytic agent by stimulating adenylate cyclase, thereby reducing intracellular lipid levels. In contrast, carnitine functions as a lipid metabolism enhancer, facilitating the transport of long-chain fatty acids into mitochondria for β-oxidation and promoting mitochondrial energy metabolism [145,146]. However, several studies have also indicated that supplementation with fatty acids during the vitrification of mouse oocytes or 4-cell embryos can increase neutral lipid content in mouse oocytes, improve the blastocyst development rate, and upregulate the expression of genes related to fatty acid β-oxidation in mouse embryos [147]. Fatty acid β-oxidation is a metabolic process that occurs within mitochondria, in which fatty acids are broken down into acetyl-CoA. This process promotes ATP production via the electron transport chain [148,149], thereby supporting cellular energy metabolism after thawing. Therefore, the use of small-molecule lipid-reducing agents (Forskolin, levocarnitine) is a promising approach to enhance vitrification outcomes.

**Table 2:** The effects of antioxidant supplementation on the vitrification of mammalian oocytes or embryos.

Antioxidants	Research Subject	Dose	Research Findings	Reference
Ascorbic acid	Mouse 2-cell embryos or blastocysts	0.1 mM	Ascorbic acid reduces hydrogen peroxide levels in embryos, enhances inner cell mass development, and decreases lactate dehydrogenase activity.	[150]
Melatonin	Human oocytes	10^−9^ M	Addition of 10^−9^ M melatonin during human oocyte vitrification significantly reduces intracellular ROS and Ca^2+^ levels, maintains membrane integrity, and mitigates oxidative stress.	[151]
Astaxanthin	Porcine oocytes	2.5 μM	Astaxanthin improves the survival rate of vitrified oocytes, reduces ROS levels, increases glutathione levels, and enhances lysosomal fluorescence intensity.	[152]
mitoquinone (MtQ)	Mouse Oocytes	0.02 μM	MtQ improves post-warming survival, enhances MMP, and reduces the Bax/Bcl2 ratio and caspase3 expression.	[153]
L-carnitine	Porcine oocytes	10 mM	L-carnitine significantly reduces lipid droplet content and restores *SOD*1 expression in vitrified oocytes; however, it does not improve overall cryopreservation outcome.	[154]
Resveratrol	porcine oocytes	2 μM	Resveratrol improves the quality of vitrified porcine oocytes and regulates apoptosis.	[11]
Chlorogenic Acid	Ovine oocytes	40 μM	Chlorogenic acid mitigates oxidative stress, enhances mitochondrial function, and downregulates the expression of apoptosis and antioxidant-related genes in vitrified oocytes.	[17]
Resveratrol	Bovine oocytes	1 μM	Resveratrol reduces the incidence of abnormal spindles and lowers ROS levels in vitrified oocytes to physiological ranges, thereby enhancing cryopreservation efficiency.	[155]
Spermidine	Mouse oocytes	50 μM	Spermidine improves the survival and blastocyst formation rates of vitrified mouse oocytes and restores the expression levels of 43.3% of dysregulated genes.	[23]

### 6.5. Stabilizing the Cytoskeleton

The addition of cytoskeletal stabilizers during oocyte maturation has been widely reported to improve cryosurvival by maintaining cytoskeletal integrity. Commonly used stabilizers include cytochalasin B (CB), cytochalasin D, and paclitaxel. Pretreating buffalo oocytes with 8 μg/mL cytochalasin B before vitrification attenuated the reduction in tubulin expression and improved development to the 8-cell and blastocyst stages [156]. Moawad et al. [157] found that treating sheep GV oocytes with 7.5 μg/mL CB prior to vitrification improved post-warming cleavage rates.

Based on the cryotop vitrification of mouse oocytes, supplementation with docetaxel alleviates vitrification-induced damage by modulating the expression of apoptosis-related genes [158]. Existing studies have demonstrated that the addition of cytoskeletal stabilizers (e.g., cytochalasin B, docetaxel, etc.) during oocyte maturation can improve their survival rate and subsequent developmental potential by maintaining cytoskeletal integrity and regulating the expression of apoptosis-related genes [159], with the efficacy varying across different species and stabilizer concentrations. Future research should prioritize the development of novel, low-toxicity cytoskeletal stabilizers and integrate multi-omics frameworks to delineate their underlying molecular mechanisms. Such efforts are essential for the precise optimization of oocyte cryopreservation protocols across diverse species.

### 6.6. Application of Novel Biosynthetic Materials

Over the years, novel biosynthetic materials, such as nanomaterials, have been widely applied in oocyte vitrification. Importantly, novel ice-inhibiting nanomaterials, including hydroxyapatite (HA) nanoparticles, possess excellent biocompatibility, antioxidant properties, thermal conductivity, and membrane permeability. Studies have found that the addition of HA nanoparticles to vitrification solutions can significantly improve the cryosurvival rate of porcine MII oocytes reported that the survival rate increased from 14.7% to 25.9–35.4% [160]; its protective mechanism does not rely on enhancing cooling rates but rather on inhibiting ice crystal formation and recrystallization, thereby alleviating mechanical damage to cells during the freezing–thawing process [160,161]. Liu et al. [162] reported that during the vitrification of ovine oocytes using cryotop devices, supplementation with HA nanoparticles reduced ROS and apoptosis levels, increased cleavage rates, and restored MMP post-vitrification. Studies have shown that nanoparticles can scavenge intracellular oxygen-free radicals, reduce intracellular ROS levels, modulate MMP levels, and alleviate oxidative stress [163,164]. In addition, antioxidant nanomaterials, such as Fe_3_O_4_ magnetic nanoparticles and nanocarriers loaded with resveratrol [13] or melatonin [165,166] have been shown to alleviate oxidative stress during cryopreservation. Fe_3_O_4_ nanoparticles significantly improved nuclear maturation and embryonic development in mouse immature oocytes [167]. These findings collectively indicate that novel biomaterials have extremely broad application prospects in the vitrification of mammalian oocytes and are highly beneficial for improving oocyte vitrification efficiency.

### 6.7. Development of Novel Vitrification Devices

With the continuous advancement in science and technology, emerging vitrification devices have been increasingly developed and applied to oocyte vitrification preservation. Researchers have successively developed automated devices for oocyte or embryo vitrification that help mitigate osmotic damage during CPA loading [168,169]. Secondly, the gradual progression of ultra-rapid vitrification has opened up a novel avenue for oocyte vitrification. Ultra-rapid vitrification minimizes CPA volume and attenuates its cytotoxicity [56], thereby improving the post-vitrification survival rate of oocytes.

**Figure 3:**
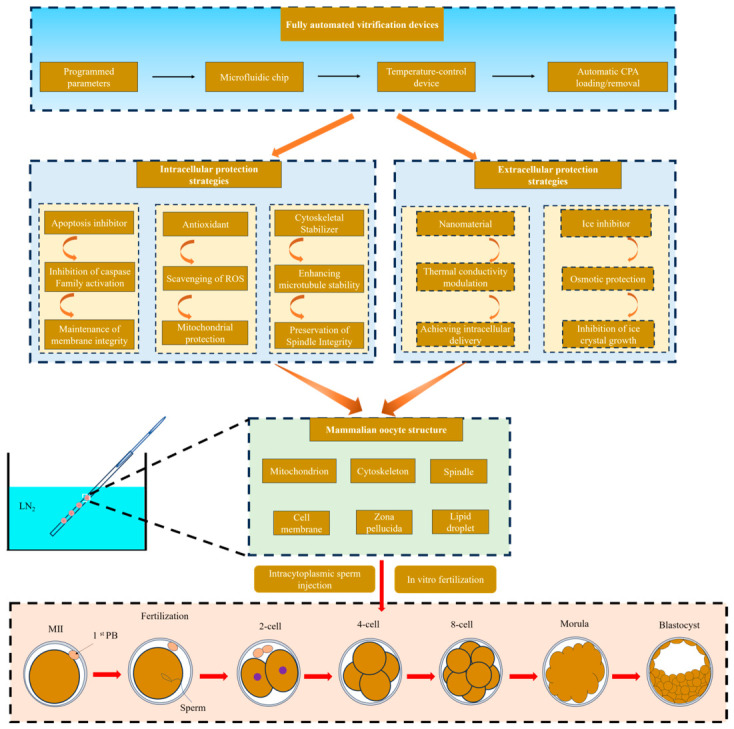
A schematic diagram of combined strategies for enhancing the developmental potential of vitrified oocytes. The integrated approach combines fully automated vitrification devices with targeted intracellular and extracellular protective strategies to mitigate cryodamage, ultimately enhancing oocyte viability and post-thaw embryonic development rates following in vitro fertilization/intracytoplasmic sperm injection.

The integrated application of these strategies significantly improves the efficiency of oocyte vitrification in mammalian species (Figure 3). Furthermore, the implementation of fully automated vitrification systems helps to minimize cellular or embryonic damage induced by manual manipulation. Concurrently, as artificial intelligence continues to advance, vitrification robots specifically engineered for mammalian oocytes or embryos and powered by large language models are anticipated to catalyze an industrial transformation in cryobiology.

## 7. Comparative Analysis of Oocyte Vitrification Across Different Species

**Figure 4:**
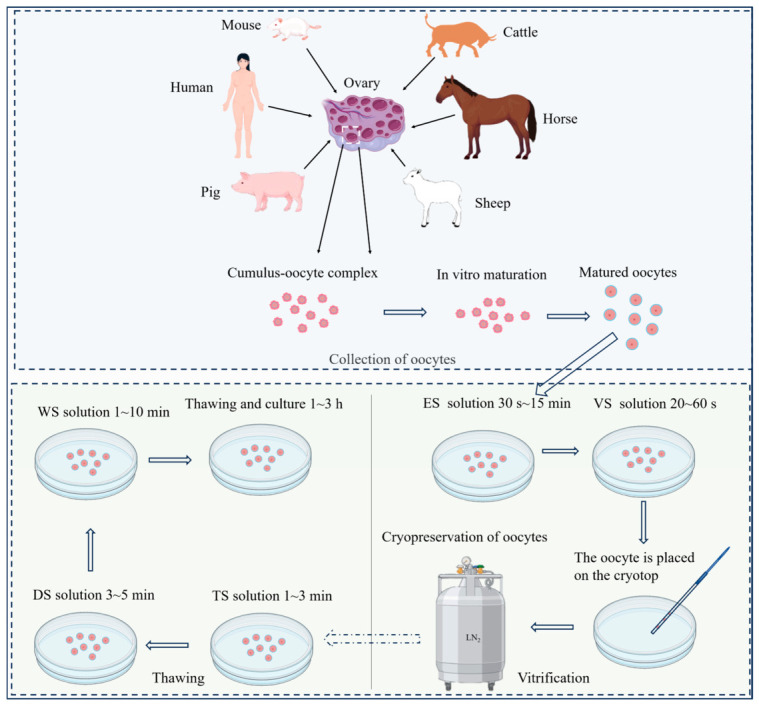
Operational procedure of mammalian oocyte vitrification and thawing. Following in vitro maturation, oocytes are generally equilibrated in equilibration solution (ES) for 30 s ~15 min, and then exposed to vitrification solution (VS) for 20~60 s, loaded onto a cryotop, and rapidly plunged into liquid nitrogen. During warming, oocytes are sequentially transferred to a trehalose/sucrose (TS) solution for 1~3 min, followed by a dilution solution (DS) for 3~5 min, and a wash solution (WS) for 1~10 min, before being placed into recovery medium for 1~3 h.

The vitrification process for oocytes from different species shares a common procedural framework consisting of three key steps: equilibration, vitrification, and warming [108,140]. During equilibration, oocytes are briefly exposed to an equilibration solution (ES) containing permeating CPAs (typically EG and DMSO), then transferred to a vitrification solution (VS) with higher concentrations of these permeating CPAs combined with non-permeating CPAs such as sucrose or trehalose to induce rapid dehydration [86,112]. Subsequently, oocytes are loaded into an ultra-minimal volume (<1 μL) on a vitrification device and immediately plunged into liquid nitrogen for storage; warming typically involves stepwise sucrose dilution to alleviate osmotic stress [86]. Due to interspecies variations in oocyte physiology, the optimal CPA composition and equilibration duration differ markedly. In mice, oocytes are briefly equilibrated 30 s in 10% (*v*/*v*) EG and 10% (*v*/*v*) DMSO, followed by a short exposure of 25 s to 15% (*v*/*v*) of EG and DMSO with sucrose [13,170]. In humans, equilibration is markedly longer, typically around 15 min in 7.5% EG and 7.5% propanediol (PROH), prior to transfer into a VS supplemented with sucrose or trehalose and serum substitutes, with stepwise sucrose dilution during warming [25]. In livestock species, protocol parameters are further adjusted. Ovine oocytes typically require intermediate equilibration of about 1 min and exposure to higher cryoprotectant concentrations [171], whereas bovine oocytes show protocol variability depending on the carrier system (e.g., Cryotop vs. OPS), with corresponding differences in VS composition and exposure time [16]. Porcine oocytes, characterized by large size and high lipid content, require the longest equilibration time, typically 5 to 15 min, and more complex warming procedures involving higher initial sucrose concentrations and gradual dilution [11]. The general workflow for the vitrification and recovery of oocytes across diverse species is illustrated in Figure 4. These interspecies discrepancies are predominantly attributable to variations in oocyte lipid profiles and membrane composition. Phospholipids, particularly phosphatidylcholine and sphingomyelin, modulate membrane fluidity and permeability [172], thereby governing water and cryoprotectant exchange [119]. Substantial intracellular lipid levels, particularly in porcine oocytes, reduce membrane permeability and prolong equilibration requirements.

Due to differences in lipid content among oocytes of different species, their vitrification efficiency also varies [37]. With a lipid content 3–5 times that of cattle and sheep [144], porcine oocytes undergo lipid phase transitions during the freezing process, resulting in a vitrification survival rate of only 58–65%, with cleavage and blastocyst rates of 35–40% and 2–5%, respectively [18,19,20]. Mouse and human oocytes have low lipid content and smaller volumes, resulting in higher vitrification survival rates. Studies have indicated that mouse oocytes exhibit vitrification survival rates of 82–90%, cleavage rates above 59–68%, and blastocyst rates of 20–37% [22,23,24]. The survival rate, good-quality embryo rate, and blastocyst rate of vitrified human oocytes were 94–100%, 54–71%, and 31.7–34.9%, respectively [25,173]. The primary reason for these differences lies in the substantial variation in lipid composition and content among oocytes; the higher the cellular lipid content, the lower the tolerance to low temperatures. During vitrification, lipid phase transitions compromise cell membrane integrity, inducing apoptosis and cell death [118]. Additionally, the types of lipids that constitute the cell membrane influence its tolerance to low temperatures. Research has found that membranes with high cholesterol and low phospholipid content are less sensitive to temperature changes, and that the higher the cellular lipid content, the lower the tolerance to low temperatures [174]. This may account for some of the differences in vitrification efficiency across species.

## 8. Discussion

Vitrification currently represents the preferred strategy for oocyte cryopreservation, surpassing slow-cooling protocols by effectively mitigating cryoinjury and maintaining cellular architecture. This transition has solidified its role as a pivotal tool in both clinical reproductive medicine and the conservation of biodiversity via endangered species germplasm banks [1,175]. Although vitrification has been widely accepted, several controversial issues persist in its application. On one hand, the relative merits of open versus closed vitrification systems remain debated: some studies suggest that closed systems prevent liquid nitrogen-mediated pathogen contamination and offer greater safety [176], whereas others report no significant differences between the two systems in terms of post-warming survival rates or developmental competence [177]. On the other hand, there is disagreement regarding the optimal developmental stage for oocyte vitrification. The mature MII stage allows direct use for in vitro fertilization with a straightforward workflow, while the immature GV stage theoretically reduces the risk of aneuploidy by avoiding spindle depolymerization induced by cryopreservation [92,93]. These discrepancies primarily stem from heterogeneity in experimental protocols, such as the type and concentration of cryoprotectants and cooling rates, as well as species-specific factors [37,178]. Current literature lacks sufficient longitudinal data on offspring health and epigenetic risks. Moreover, the precise mechanisms underlying cryoinjury-induced ultrastructural defects, such as mitochondrial and cytoskeletal abnormalities, require further elucidation [12]. Addressing these limitations will be essential for developing species-tailored vitrification strategies and improving reproductive outcomes across diverse mammalian systems.

In the future, the adoption of vitrification instruments and automated vitrification devices will enable the precise addition and removal of cryoprotectants at the nanoscale, while uniform warming processes will facilitate the standardization of vitrification techniques. Cryoprotectant-free strategies or non-permeable cryoprotectants such as trehalose and antifreeze proteins may gradually replace sucrose, enabling cell cryopreservation without chemical toxicity and eliminating cytotoxic effects. This technology will extend beyond assisted reproduction to broader applications, including establishing “germplasm banks” for endangered species, thereby supporting biodiversity conservation. In summary, through interdisciplinary integration, oocyte vitrification is evolving toward safer, more efficient, and more intelligent approaches, opening new frontiers in reproductive medicine and species conservation.

## 9. Conclusions

Oocyte vitrification is the primary method for preserving germplasm in female animals and has been successfully applied in multiple mammalian species. It is widely recognized that the vitrification process induces osmotic stress and cryoprotectant toxicity, leading to cellular damage that compromises developmental competence. However, the molecular mechanisms underlying these injuries, particularly their long-term epigenetic consequences, remain largely unknown. Whether oxidative stress, mitochondrial dysfunction, and apoptosis occur independently or interact to cause cellular damage remains unclear. It also remains unexplored whether epigenetic alterations induced by vitrification can be transmitted to offspring and affect their health. Key priorities include decoding the molecular and epigenetic impacts of cryoinjury, standardizing oocyte vitrification through automation, and developing biocompatible cryoprotectants to ensure precise delivery and minimal toxicity. Addressing these challenges is essential for improving the efficiency of oocyte vitrification in mammals, particularly in large animals such as pigs, cattle, and sheep, for accelerating genetic improvement in farm animals, and for facilitating the preservation of germplasm resources from endangered species.

## Data Availability

The data presented in this study are available in this article.

## Abbreviations

The following abbreviations are used in this manuscript:

CPAs	Cryoprotectants
EG	Ethylene glycol
DMSO	Dimethyl sulfoxide
GV	Germinal vesicle
MII	Metaphase II
OPS	Open pulled straw
NADPH	Reduced nicotinamide adenine dinucleotide phosphate
MMP	Mitochondrial membrane potential
ATP	Adenosine triphosphate
ICSI	Intracytoplasmic sperm injection
ROS	Reactive oxygen species
SSV	Solid surface vitrification
ZP	Zona pellucida
MtQ	Mitoquinone
IGF2R	Insulin-like growth factor 2 receptor
PPP1R9A	Protein phosphatase 1 regulatory subunit 9A
PEG3	Paternally expressed gene 3
H3K9ac	Histone H3 lysine 9 acetylation
H4K5ac	Histone H4 lysine 5 acetylation
H4K21ac	Histone H4 lysine 21 acetylation
H3K9me3	H3 lysine 9 trimethylation
LDHA	Lactate dehydrogenase A
LDHA	Lactate dehydrogenase B
EP300	E1A binding protein p300
SIRT1	Sirtuin1
Gtl2	Gene trap locus 2
FfIBP	Flavobacterium frigoris ice-binding proteins
LeIBP	Glaciozyma sp. ice-binding proteins
ApAFP914	Antifreeze proteins from Anatolia polita
CB	Cytochalasin B
HA	Hydroxyapatite
Z-VAD-FMK	Benzyloxycarbonyl-Val-Ala-Asp -fluoromethyl ketone
Y-27632	(R)-(+)-trans-N-(4-pyridyl)-4-(1-aminoethyl)cyclohexanecarboxamide

## References

[1] Somfai T., Nguyen V.K., Vu H.T.T., Nguyen H.L.T., Quan H.X., Viet Linh N., Phan S.L., Pham L.D., Cuc N.T.K., Kikuchi K. (2019). Cryopreservation of immature oocytes of the indigeneous Vietnamese Ban Pig. Anim. Sci. J..

[2] Mandawala A.A., Harvey S.C., Roy T.K., Fowler K.E. (2016). Cryopreservation of animal oocytes and embryos: Current progress and future prospects. Theriogenology.

[3] Hryhorowicz M., Lipiński D., Hryhorowicz S., Nowak-Terpiłowska A., Ryczek N., Zeyland J. (2020). Application of Genetically Engineered Pigs in Biomedical Research. Genes.

[4] Prentice J.R., Anzar M. (2010). Cryopreservation of Mammalian oocyte for conservation of animal genetics. Vet. Med. Int..

[5] Gil M.A., Parrilla I., Cuello C., Martinez E.A. (2025). Current status of nonsurgical embryo transfer in swine. Reprod. Fertil. Dev..

[6] Woelders H., Windig J., Hiemstra S.J. (2012). How developments in cryobiology, reproductive technologies and conservation genomics could shape gene banking strategies for (farm) animals. Reprod. Domest. Anim..

[7] Leroy G., Boettcher P., Besbes B., Danchin-Burge C., Baumung R., Hiemstra S.J. (2019). Cryoconservation of Animal Genetic Resources in Europe and Two African Countries: A Gap Analysis. Diversity.

[8] Rall W.F., Fahy G.M. (1985). Ice-free cryopreservation of mouse embryos at -196 degrees C by vitrification. Nature.

[9] Ayantoye J.O., Yang B., Zhang H., Dong J., Zhang X., Song H., Shahzad M., Kolachi H.A., Osaiyuwu O.H., Wan P. (2026). Overcoming the warming bottleneck in animal vitrification: Volumetric heating and enabling technologies for reproductive cryobanking. Prog. Biophys. Mol. Biol..

[10] Kuleshova L., Gianaroli L., Magli C., Ferraretti A., Trounson A. (1999). Birth following vitrification of a small number of human oocytes: Case report. Hum. Reprod..

[11] Giaretta E., Spinaci M., Bucci D., Tamanini C., Galeati G. (2013). Effects of resveratrol on vitrified porcine oocytes. Oxid. Med. Cell. Longev..

[12] Zhou D., Liu H., Zheng L., Liu A., Zhuan Q., Luo Y., Zhou G., Meng L., Hou Y., Wu G. (2024). Metformin alleviates cryoinjuries in porcine oocytes by reducing membrane fluidity through the suppression of mitochondrial activity. Commun. Biol..

[13] Zhu Y., Li J., Zhou G., Zou X., Liu A., ReYiMuJiang Y., Sun Y., Luo Y., Xia H., Bai J. (2025). Resveratrol-Loaded PLGA Enhanced Vitrified Oocyte Viability through Rab11fip4/Arf5-Mediated Internalization Route. ACS Appl. Mater. Interfaces.

[14] Jia Q.P., Sun W.Q. (2021). PERSPECTIVE: Cryopreservation of Human Oocytes and the ‘Carryover’ Effect on Early Embryo Development. Cryo Lett..

[15] Xu X., Yang B., Zhang H., Feng X., Hao H., Du W., Zhu H., Khan A., Khan M.Z., Zhang P. (2023). Effects of β-Nicotinamide Mononucleotide, Berberine, and Cordycepin on Lipid Droplet Content and Developmental Ability of Vitrified Bovine Oocytes. Antioxidants.

[16] Alfradique V.A.P., Oliveira T.A., Silva T.E.C., Apolinario G.S., Batista R., Souza-Fabjan J.M.G. (2026). L-carnitine or melatonin supplementation during vitrification and warming mitigates oxidative stress and improves cryotolerance in immature bovine cumulus-oocyte complexes. Reprod. Biol..

[17] Tao H., Zhao Y., Zhang Q., Li X., Hu G., Wang Y., Zeng W. (2025). Effects of Chlorogenic Acid on In Vitro Maturation and Vitrification Cryopreservation of Sheep Oocytes. Vet. Sci..

[18] Xu J., He M., Gao J., Sun L., Wu C., Zhang S., Zhang D., Dai J. (2026). Manipulation of autophagy regulates mitochondrial homeostasis and early embryo development of cryopreserved porcine oocytes. Anim. Reprod. Sci..

[19] Vallorani C., Spinaci M., Bucci D., Porcu E., Tamanini C., Galeati G. (2012). Pig oocyte vitrification by Cryotop method and the activation of the apoptotic cascade. Anim. Reprod. Sci..

[20] Xu J., Sun L., Wu C., Zhang S., Ju S., Rui R., Zhang D., Dai J. (2021). Involvement of PINK1/Parkin-mediated mitophagy in mitochondrial functional disruption under oxidative stress in vitrified porcine oocytes. Theriogenology.

[21] Maclellan L.J., Albertini D.F., Stokes J.E., Carnevale E.M. (2023). Use of confocal microscopy and intracytoplasmic sperm injection (ICSI) to assess viability of equine oocytes from young and old mares after vitrification. J. Assist. Reprod. Genet..

[22] Deng D., Xie J., Tian Y., Zhu L., Liu X., Liu J., Huang G., Li J. (2023). Effects of meiotic stage-specific oocyte vitrification on mouse oocyte quality and developmental competence. Front. Endocrinol..

[23] Wang L., Li W., Liu Y., Dilixiati A., Chang Z., Liang Y., Wang Y., Ma X., Tang L., He Z. (2025). Spermidine Supplementation Effectively Improves the Quality of Mouse Oocytes After Vitrification Freezing. Antioxidants.

[24] Pan B., Qazi I.H., Guo S., Yang J., Qin J., Lv T., Zang S., Zhang Y., Zeng C., Meng Q. (2021). Melatonin improves the first cleavage of parthenogenetic embryos from vitrified-warmed mouse oocytes potentially by promoting cell cycle progression. J. Anim. Sci. Biotechnol..

[25] Zhang Z., Wang T., Hao Y., Panhwar F., Chen Z., Zou W., Ji D., Chen B., Zhou P., Zhao G. (2017). Effects of trehalose vitrification and artificial oocyte activation on the development competence of human immature oocytes. Cryobiology.

[26] Chang C.C., Shapiro D.B., Nagy Z.P. (2021). The effects of vitrification on oocyte quality. Biol. Reprod..

[27] Marteil G., Metchat A., Dollet S., Cugnot C., Chaput L., Pereira B., Gremeau A.S., Brugnon F. (2024). Vitrification of Human Oocytes Before or After Rescue-IVM Does not Impair Maturation Kinetics but Induces Meiotic Spindle Alterations. Reprod. Sci..

[28] Arav A., Zvi R. (2008). Do chilling injury and heat stress share the same mechanism of injury in oocytes?. Mol. Cell. Endocrinol..

[29] Zhu Y., Liu H., Zheng L., Luo Y., Zhou G., Li J., Hou Y., Fu X. (2024). Vitrification of Mammalian Oocytes: Recent Studies on Mitochondrial Dysfunction. Biopreserv. Biobank.

[30] López A., Ducolomb Y., Casas E., Retana-Márquez S., Betancourt M., Casillas F. (2021). Effects of Porcine Immature Oocyte Vitrification on Actin Microfilament Distribution and Chromatin Integrity During Early Embryo Development in vitro. Front. Cell Dev. Biol..

[31] Wang Y., Chang H., He Q., Xue Y., Zhang K., Kang J., Wang Y., Xu Z., Zhang Y., Quan F. (2021). Effect of oocyte vitrification on glucose transport in mouse metaphase II oocytes. Reproduction.

[32] Wei X., Sijie Y., Weibin Z., Qing X., Jie Z., Xiangdong Z. (2018). Cytoskeleton Genes Expression and Survival Rate Comparison Between Immature and Mature Yak Oocyte After OPS Vitrification. Anim. Biotechnol..

[33] Tatone C., Di Emidio G., Vento M., Ciriminna R., Artini P.G. (2010). Cryopreservation and oxidative stress in reproductive cells. Gynecol. Endocrinol..

[34] Manna C., Rienzi L., Greco E., Sbracia M., Rahman A., Poverini R., Siracusa G., De Felici M. (2001). Zona pellucida solubility and cortical granule complements in human oocytes following assisted reproductive techniques. Zygote.

[35] Gao H.-H., Li J.-T., Liu J.-J., Yang Q.-A., Zhang J.-M. (2017). Autophagy inhibition of immature oocytes during vitrification-warming and in vitro mature activates apoptosis via caspase-9 and-12 pathway. Eur. J. Obstet. Gynecol. Reprod. Biol..

[36] Spinaci M., Vallorani C., Bucci D., Tamanini C., Porcu E., Galeati G. (2012). Vitrification of pig oocytes induces changes in histone H4 acetylation and histone H3 lysine 9 methylation (H3K9). Vet. Res. Commun..

[37] Olexiková L., Makarevich A., Dujíčková L., Kubovičová E., Chrenek P. (2024). Factors affecting cryotolerance of mammalian oocytes. Cryobiology.

[38] Viana I.G.R., Vireque A.A., Navarro P.A. (2022). Comparing the effects of a commercial and a prototype vitrification medium on meiotic spindle morphology and survival rate of mouse oocytes. JBRA Assist. Reprod..

[39] Bianchi V., Macchiarelli G., Borini A., Lappi M., Cecconi S., Miglietta S., Familiari G., Nottola S.A. (2014). Fine morphological assessment of quality of human mature oocytes after slow freezing or vitrification with a closed device: A comparative analysis. Reprod. Biol. Endocrinol..

[40] Ma Y., Gao L., Tian Y., Chen P., Yang J., Zhang L. (2021). Advanced biomaterials in cell preservation: Hypothermic preservation and cryopreservation. Acta Biomater..

[41] Iussig B., Maggiulli R., Fabozzi G., Bertelle S., Vaiarelli A., Cimadomo D., Ubaldi F.M., Rienzi L. (2019). A brief history of oocyte cryopreservation: Arguments and facts. Acta Obstet. Gynecol. Scand..

[42] Gupta M.K., Uhm S.J., Lee H.T. (2010). Effect of vitrification and beta-mercaptoethanol on reactive oxygen species activity and in vitro development of oocytes vitrified before or after in vitro fertilization. Fertil. Steril..

[43] Zhang X., Wu X.Q., Lu S., Guo Y.L., Ma X. (2006). Deficit of mitochondria-derived ATP during oxidative stress impairs mouse MII oocyte spindles. Cell Res..

[44] Rusciano G., De Canditiis C., Zito G., Rubessa M., Roca M.S., Carotenuto R., Sasso A., Gasparrini B. (2017). Raman-microscopy investigation of vitrification-induced structural damages in mature bovine oocytes. PLoS ONE.

[45] Succu S., Gadau S.D., Serra E., Zinellu A., Carru C., Porcu C., Naitana S., Berlinguer F., Leoni G.G. (2018). A recovery time after warming restores mitochondrial function and improves developmental competence of vitrified ovine oocytes. Theriogenology.

[46] Agarwal A., Sharma R.K., Nallella K.P., Thomas A.J., Alvarez J.G., Sikka S.C. (2006). Reactive oxygen species as an independent marker of male factor infertility. Fertil. Steril..

[47] Mateo-Otero Y., Yeste M., Damato A., Giaretta E. (2021). Cryopreservation and oxidative stress in porcine oocytes. Res. Vet. Sci..

[48] Dai J., Wu C., Muneri C.W., Niu Y., Zhang S., Rui R., Zhang D. (2015). Changes in mitochondrial function in porcine vitrified MII-stage oocytes and their impacts on apoptosis and developmental ability. Cryobiology.

[49] Thomas C., Mackey M.M., Diaz A.A., Cox D.P. (2009). Hydroxyl radical is produced via the Fenton reaction in submitochondrial particles under oxidative stress: Implications for diseases associated with iron accumulation. Redox Rep..

[50] Guo X.F., Yu X.L., Zhang F., Wu H., Pei X.Z., Li X.X., Li Y.H. (2017). Effect of liquid helium vitrification on cytoskeleton of immature cattle oocytes. Anim. Reprod. Sci..

[51] García-Martínez T., Vendrell-Flotats M., Martínez-Rodero I., Ordóñez-León E.A., Álvarez-Rodríguez M., López-Béjar M., Yeste M., Mogas T. (2020). Glutathione Ethyl Ester Protects In Vitro—Maturing Bovine Oocytes against Oxidative Stress Induced by Subsequent Vitrification/Warming. Int. J. Mol. Sci..

[52] Wang W.H., Meng L., Hackett R.J., Odenbourg R., Keefe D.L. (2001). Limited recovery of meiotic spindles in living human oocytes after cooling-rewarming observed using polarized light microscopy. Hum. Reprod..

[53] Guo Y., Sun H., Chen H., Yang G., Wang J., Qi Z., Pang W., Chu G., Gao L. (2023). Vitrification induces a focused spindle pole in mouse MI oocytes. Theriogenology.

[54] Prosser S.L., Pelletier L. (2017). Mitotic spindle assembly in animal cells: A fine balancing act. Nat. Rev. Mol. Cell Biol..

[55] Severance A.L., Latham K.E. (2018). Meeting the meiotic challenge: Specializations in mammalian oocyte spindle formation. Mol. Reprod. Dev..

[56] Cho J.R., Yu E.H., Lee H.J., Kim I.H., Jeong J.H., Lee D.B., Cho S.K., Joo J.K. (2024). Ultra-Fast Vitrification: Minimizing the Toxicity of Cryoprotective Agents and Osmotic Stress in Mouse Oocyte Cryopreservation. Int. J. Mol. Sci..

[57] Shin H., Song H., Suh C.S., Lim H.J. (2013). The formin protein mDia2 serves as a marker of spindle pole dynamics in vitrified-warmed mouse oocytes. PLoS ONE.

[58] Balboula A.Z., Schindler K., Kotani T., Kawahara M., Takahashi M. (2020). Vitrification-induced activation of lysosomal cathepsin B perturbs spindle assembly checkpoint function in mouse oocytes. Mol. Hum. Reprod..

[59] Boyman L., Karbowski M., Lederer W.J. (2020). Regulation of Mitochondrial ATP Production: Ca 2+ Signaling and Quality Control. Trends Mol. Med..

[60] Brookes P.S., Yoon Y., Robotham J.L., Anders M.W., Sheu S.S. (2004). Calcium, ATP, and ROS: A mitochondrial love-hate triangle. Am. J. Physiol. Cell Physiol..

[61] Shoshan-Barmatz V., Krelin Y., Shteinfer-Kuzmine A. (2018). VDAC1 functions in Ca 2+ homeostasis and cell life and death in health and disease. Cell Calcium.

[62] Vercellino I., Sazanov L.A. (2021). The assembly, regulation and function of the mitochondrial respiratory chain. Nat. Rev. Mol. Cell Biol..

[63] Zhao R.Z., Jiang S., Zhang L., Yu Z.B. (2019). Mitochondrial electron transport chain, ROS generation and uncoupling (Review). Int. J. Mol. Med..

[64] Amoushahi M., Salehnia M., Mowla S.J. (2017). Vitrification of Mouse MII Oocyte Decreases the Mitochondrial DNA Copy Number, TFAM Gene Expression and Mitochondrial Enzyme Activity. J. Reprod. Infertil..

[65] Lei T., Guo N., Tan M.H., Li Y.F. (2014). Effect of mouse oocyte vitrification on mitochondrial membrane potential and distribution. J. Huazhong Univ. Sci. Technol. Med. Sci..

[66] Gutierrez-Castillo E., Diaz F.A., Talbot S.A., Bondioli K.R. (2022). Recovery of spindle morphology and mitochondrial function through extended culture after vitrification-warming of bovine oocytes. Theriogenology.

[67] Morado S., Aparicio A., Pinchetti D., Arraztoa C.C., Alvarez G., Gutnisky C., Neild D., Dalvit G., Cetica P. (2023). Variations in metabolic parameters of in vitro matured porcine oocytes after vitrification-warming. Open Vet. J..

[68] Abd El-Nasser Ahmed M., Al-Suwaiegh S., AlGherair I. (2024). An Insight into the Roles of Zona Pellucida in Growth and Development of Mammalian Ocytes and Embryos: Changes of Age-related and Cryopreservation: A Review. Indian J. Anim. Res..

[69] Choi J.K., Yue T., Huang H., Zhao G., Zhang M., He X. (2015). The crucial role of zona pellucida in cryopreservation of oocytes by vitrification. Cryobiology.

[70] Ghetler Y., Skutelsky E., Ben Nun I., Ben Dor L., Amihai D., Shalgi R. (2006). Human oocyte cryopreservation and the fate of cortical granules. Fertil. Steril..

[71] Nottola S.A., Albani E., Coticchio G., Palmerini M.G., Lorenzo C., Scaravelli G., Borini A., Levi-Setti P.E., Macchiarelli G. (2016). Freeze/thaw stress induces organelle remodeling and membrane recycling in cryopreserved human mature oocytes. J. Assist. Reprod. Genet..

[72] Tiwari M., Prasad S., Tripathi A., Pandey A.N., Ali I., Singh A.K., Shrivastav T.G., Chaube S.K. (2015). Apoptosis in mammalian oocytes: A review. Apoptosis.

[73] Tripathi A., Shrivastav T.G., Chaube S.K. (2013). An increase of granulosa cell apoptosis mediates aqueous neem (Azadirachta indica) leaf extract-induced oocyte apoptosis in rat. Int. J. Appl. Basic. Med. Res..

[74] Chaube S.K., Shrivastav T.G., Tiwari M., Prasad S., Tripathi A., Pandey A.K. (2014). Neem leaf extract deteriorates oocyte quality by inducing ROS-mediated apoptosis in mammals. Springerplus.

[75] Chaube S.K., Prasad P.V., Thakur S.C., Shrivastav T.G. (2005). Hydrogen peroxide modulates meiotic cell cycle and induces morphological features characteristic of apoptosis in rat oocytes cultured in vitro. Apoptosis.

[76] McConkey D.J., Orrenius S. (1997). The role of calcium in the regulation of apoptosis. Biochem. Biophys. Res. Commun..

[77] Tiwari M., Prasad S., Shrivastav T.G., Chaube S.K. (2017). Calcium Signaling During Meiotic Cell Cycle Regulation and Apoptosis in Mammalian Oocytes. J. Cell. Physiol..

[78] Jurisicova A., Acton B.M. (2004). Deadly decisions: The role of genes regulating programmed cell death in human preimplantation embryo development. Reproduction.

[79] Liu L., Trimarchi J.R., Keefe D.L. (2000). Involvement of mitochondria in oxidative stress-induced cell death in mouse zygotes. Biol. Reprod..

[80] Zhao Y.H., Wang J.J., Zhang P.P., Hao H.S., Pang Y.W., Wang H.Y., Du W.H., Zhao S.J., Ruan W.M., Zou H.Y. (2020). Oocyte IVM or vitrification significantly impairs DNA methylation patterns in blastocysts as analysed by single-cell whole-genome methylation sequencing. Reprod. Fertil. Dev..

[81] Wei Y., Pan B., Qin J., Cao B., Lv T., Ye J., Ning A., Du K., Chen X., Zou S. (2024). The walnut-derived peptide TW-7 improves mouse parthenogenetic embryo development of vitrified MII oocytes potentially by promoting histone lactylation. J. Anim. Sci. Biotechnol..

[82] Chen H., Zhang L., Wang Z., Chang H., Xie X., Fu L., Zhang Y., Quan F. (2019). Resveratrol improved the developmental potential of oocytes after vitrification by modifying the epigenetics. Mol. Reprod. Dev..

[83] Afzalnia S., Ghasemian F., Maryan H.S., Mirzapour T. (2025). Ultrastructure assessment of oocyte from mouse with polycystic ovary syndrome following cryopreservation. Cryo Lett..

[84] Li X., Wang Y.K., Song Z.Q., Du Z.Q., Yang C.X. (2016). Dimethyl Sulfoxide Perturbs Cell Cycle Progression and Spindle Organization in Porcine Meiotic Oocytes. PLoS ONE.

[85] Berlinguer F., Succu S., Mossa F., Madeddu M., Bebbere D., Leoni G.G., Naitana S. (2007). Effects of trehalose co-incubation on in vitro matured prepubertal ovine oocyte vitrification. Cryobiology.

[86] Kuwayama M. (2007). Highly efficient vitrification for cryopreservation of human oocytes and embryos: The Cryotop method. Theriogenology.

[87] Quan G., Wu G., Hong Q. (2017). Oocyte Cryopreservation Based in Sheep: The Current Status and Future Perspective. Biopreserv. Biobank.

[88] Yu X.L., Xu Y.K., Wu H., Guo X.F., Li X.X., Han W.X., Li Y.H. (2016). Successful vitrification of bovine immature oocyte using liquid helium instead of liquid nitrogen as cryogenic liquid. Theriogenology.

[89] Liebermann J., Dietl J., Vanderzwalmen P., Tucker M.J. (2003). Recent developments in human oocyte, embryo and blastocyst vitrification: Where are we now?. Reprod. BioMed. Online.

[90] Kochan J., Nowak A., Młodawska W., Prochowska S., Partyka A., Skotnicki J., Niżański W. (2020). Comparison of the Morphology and Developmental Potential of Oocytes Obtained from Prepubertal and Adult Domestic and Wild Cats. Anim. Open Access J..

[91] Daddangadi A., Uppangala S., Kalthur G., Talevi R., Adiga S.K. (2020). Germinal stage vitrification is superior to MII stage vitrification in prepubertal mouse oocytes. Cryobiology.

[92] Holubcova Z., Kyjovská D., Otevřel P., Kloudová S. (2023). P-248 The meiotic spindle is preserved during freezing but post-thawing rehydration causes its destabilization in human eggs. Hum. Reprod..

[93] Sun H., Guo Y., Yu R., Wang J., Liu Y., Chen H., Pang W., Yang G., Chu G., Gao L. (2023). Ru360 protects against vitrification-induced oocyte meiotic defects by restoring mitochondrial function. Theriogenology.

[94] Jiao G.Z., Cao X.Y., Cui W., Lian H.Y., Miao Y.L., Wu X.F., Han D., Tan J.H. (2013). Developmental potential of prepubertal mouse oocytes is compromised due mainly to their impaired synthesis of glutathione. PLoS ONE.

[95] Leoni G.G., Palmerini M.G., Satta V., Succu S., Pasciu V., Zinellu A., Carru C., Macchiarelli G., Nottola S.A., Naitana S. (2015). Differences in the Kinetic of the First Meiotic Division and in Active Mitochondrial Distribution between Prepubertal and Adult Oocytes Mirror Differences in their Developmental Competence in a Sheep Model. PLoS ONE.

[96] Lee E., Baiz C.R. (2022). How cryoprotectants work: Hydrogen-bonding in low-temperature vitrified solutions. Chem. Sci..

[97] Brewer A., Girka E., Dalton A., Gutierrez-Castillo E., Bondioli K.R. (2025). Bovine oocyte vitrification and recovery with ethylene glycol and either propylene glycol or dimethyl sulfoxide. Theriogenology.

[98] Verheijen M., Lienhard M., Schrooders Y., Clayton O., Nudischer R., Boerno S., Timmermann B., Selevsek N., Schlapbach R., Gmuender H. (2019). DMSO induces drastic changes in human cellular processes and epigenetic landscape in vitro. Sci. Rep..

[99] Zhao J., Tian H., Kong X., Dang D., Liu K., Su C., Lian H., Gao T., Fu T., Zhang L. (2025). Microbiomic and Metabolomic Insights into the Mechanisms of Alfalfa Polysaccharides and Seaweed Polysaccharides in Alleviating Diarrhea in Pre-Weaning Holstein Calves. Anim. Open Access J..

[100] Papis K. (2026). A brief overview of the development of oocyte and embryo cryopreservation strategies with a focus on the roles of sugars. Cryobiology.

[101] Tsai S., Yen W., Chavanich S., Viyakarn V., Lin C. (2015). Development of Cryopreservation Techniques for Gorgonian (Junceella juncea) Oocytes through Vitrification. PLoS ONE.

[102] Huang W.-J., Zhang D., Hong Z.-W., Chen Z.-B., Dong L.-H., Zhang Y., Chen G.-Y., Liu Y., Yao B. (2020). Sequential interval micro-droplet loading in closed hemi-straw carrier system: A convenient and efficient method for ultra-rapid cryopreservation in extreme oligozoospermia. Cryobiology.

[103] Hochi S. (2022). Cryodevices developed for minimum volume cooling vitrification of bovine oocytes. Anim. Sci. J..

[104] Zhang X., Catalano P.N., Gurkan U.A., Khimji I., Demirci U. (2011). Emerging technologies in medical applications of minimum volume vitrification. Nanomedicine.

[105] Miao S., Guo C., Jiang Z., Wei H.X., Jiang X., Gu J., Hai Z., Wang T., Liu Y.H. (2022). Development of an Open Microfluidic Platform for Oocyte One-Stop Vitrification with Cryotop Method. Biosensors.

[106] Yagoub S.H., Lim M., Tan T.C.Y., Chow D.J.X., Dholakia K., Gibson B.C., Thompson J.G., Dunning K.R. (2022). Vitrification within a nanoliter volume: Oocyte and embryo cryopreservation within a 3D photopolymerized device. J. Assist. Reprod. Genet..

[107] Shen J., Yu Z., Li W., Zhou X. (2025). Oocytes Vitrification Using Automated Equipment Based on Microfluidic Chip. Ann. Biomed. Eng..

[108] He X., Park E.Y., Fowler A., Yarmush M.L., Toner M. (2008). Vitrification by ultra-fast cooling at a low concentration of cryoprotectants in a quartz micro-capillary: A study using murine embryonic stem cells. Cryobiology.

[109] Sansinena M., Santos M.V., Zaritzky N., Chirife J. (2011). Numerical simulation of cooling rates in vitrification systems used for oocyte cryopreservation. Cryobiology.

[110] Ebrahimi B., Valojerdi M.R., Eftekhari-Yazdi P., Baharvand H., Farrokhi A. (2010). IVM and gene expression of sheep cumulus-oocyte complexes following different methods of vitrification. Reprod. BioMed. Online.

[111] Berteli T.S., Vireque A.A., Luz C.M.D., Borges E.D., Ferreira C.R., Navarro P.A. (2022). Equilibration solution composition and extended exposure to equilibration phase affect embryo development and lipid profile of mouse oocytes. Reprod. BioMed. Online.

[112] García-Martínez T., Martínez-Rodero I., Roncero-Carol J., Yánez-Ortiz I., Higgins A.Z., Mogas T. (2022). Impact of equilibration duration combined with temperature on the outcome of bovine oocyte vitrification. Theriogenology.

[113] Quan G., Lv C., Liang J., Zhao X., Wu G. (2022). The Assessment of Various Factors Affecting the Postwarming Viability and Developmental Capability of Goat Metaphase II Oocytes Vitrified by Cryotop. Biopreserv. Biobank.

[114] Shirazi A., Taheri F., Nazari H., Norbakhsh-nia M., Ahmadi E., Heidari B. (2014). Developmental competence of ovine oocyte following vitrification: Effect of oocyte developmental stage, cumulus cells, cytoskeleton stabiliser, FBS concentration; equilibration time. Zygote.

[115] Paynter S.J., O’Neil L., Fuller B.J., Shaw R.W. (2001). Membrane permeability of human oocytes in the presence of the cryoprotectant propane-1,2-diol. Fertil. Steril..

[116] Szurek E.A., Eroglu A. (2011). Comparison and avoidance of toxicity of penetrating cryoprotectants. PLoS ONE.

[117] Amstislavsky S., Mokrousova V., Brusentsev E., Okotrub K., Comizzoli P. (2019). Influence of Cellular Lipids on Cryopreservation of Mammalian Oocytes and Preimplantation Embryos: A Review. Biopreserv. Biobank.

[118] Quinn P.J. (1985). A lipid-phase separation model of low-temperature damage to biological membranes. Cryobiology.

[119] Horvath G., Seidel G.E. (2006). Vitrification of bovine oocytes after treatment with cholesterol-loaded methyl-beta-cyclodextrin. Theriogenology.

[120] Isachenko V., Isachenko E., Michelmann H.W., Alabart J.L., Vazquez I., Bezugly N., Nawroth F. (2001). Lipolysis and ultrastructural changes of intracellular lipid vesicles after cooling of bovine and porcine GV-oocytes. Anat. Histol. Embryol..

[121] Ren L., Fu B., Ma H., Liu D. (2015). Effects of mechanical delipation in porcine oocytes on mitochondrial distribution, ROS activity and viability after vitrification. Cryo Lett..

[122] Moawad A.R., Zhu J., Choi I., Amarnath D., Chen W., Campbell K.H. (2013). Production of good-quality blastocyst embryos following IVF of ovine oocytes vitrified at the germinal vesicle stage using a cryoloop. Reprod. Fertil. Dev..

[123] Vajta G., Holm P., Kuwayama M., Booth P.J., Jacobsen H., Greve T., Callesen H. (1998). Open Pulled Straw (OPS) vitrification: A new way to reduce cryoinjuries of bovine ova and embryos. Mol. Reprod. Dev..

[124] Somfai T., Kikuchi K. (2021). Vitrification of Porcine Oocytes and Zygotes in Microdrops on a Solid Metal Surface or Liquid Nitrogen. Methods Mol. Biol..

[125] Olexiková L., Dujíčková L., Kubovičová E., Pivko J., Chrenek P., Makarevich A.V. (2020). Development and ultrastructure of bovine matured oocytes vitrified using electron microscopy grids. Theriogenology.

[126] Hsu J., Fatuzzo N., Weng N., Michno W., Dong W., Kienle M., Dai Y., Pasca A., Abu-Remaileh M., Rasgon N. (2023). Carnitine octanoyltransferase is important for the assimilation of exogenous acetyl-L-carnitine into acetyl-CoA in mammalian cells. J. Biol. Chem..

[127] Viana I.G.R., Vireque A.A., Da Luz C.M., Alberici L.C., Navarro P.A. (2026). Fatty acids and L-carnitine supplementation in vitrification media improves oocyte mitochondrial function and inner cell mass in mouse blastocysts: A pilot study. J. Assist. Reprod. Genet..

[128] Fang L., Bai C., Chen Y., Dai J., Xiang Y., Ji X., Huang C., Dong Q. (2014). Inhibition of ROS production through mitochondria-targeted antioxidant and mitochondrial uncoupling increases post-thaw sperm viability in yellow catfish. Cryobiology.

[129] Marthandan S., Murphy M.P., Billett E., Barnett Y. (2011). An investigation of the effects of MitoQ on human peripheral mononuclear cells. Free Radic. Res..

[130] Pervaiz S., Holme A.L. (2009). Resveratrol: Its biologic targets and functional activity. Antioxid. Redox Signal.

[131] Speranza L., Pesce M., Patruno A., Franceschelli S., Lutiis M.A., Grilli A., Felaco M. (2012). Astaxanthin treatment reduced oxidative induced pro-inflammatory cytokines secretion in U937: SHP-1 as a novel biological target. Mar. Drugs.

[132] Zhang C., Kang X., Zhang T., Huang J. (2019). Positive Effects of Resveratrol on Egg-Laying Ability, Egg Quality, and Antioxidant Activity in Hens. J. Appl. Poult. Res..

[133] Davies P.L. (2014). Ice-binding proteins: A remarkable diversity of structures for stopping and starting ice growth. Trends Biochem. Sci..

[134] Pal P., Aich R., Chakraborty S., Jana B. (2022). Molecular Factors of Ice Growth Inhibition for Hyperactive and Globular Antifreeze Proteins: Insights from Molecular Dynamics Simulation. Langmuir.

[135] Lee H.H., Lee H.J., Kim H.J., Lee J.H., Ko Y., Kim S.M., Lee J.R., Suh C.S., Kim S.H. (2015). Effects of antifreeze proteins on the vitrification of mouse oocytes: Comparison of three different antifreeze proteins. Hum. Reprod..

[136] Li X., Wang L., Yin C., Lin J., Wu Y., Chen D., Qiu C., Jia B., Huang J., Jiang X. (2020). Antifreeze protein from Anatolia polita (ApAFP914) improved outcome of vitrified in vitro sheep embryos. Cryobiology.

[137] Leal G.R., Prellwitz L., Correia L.F.L., Oliveira T.A., Guimarães M.P.P., Xavier-Getirana B.R., Dias Â.J.B., Batista R., Souza-Fabjan J.M.G. (2024). Antifreeze protein type I in the vitrification solution improves the cryopreservation of immature cat oocytes. Theriogenology.

[138] Santos E., Somfai T., Appeltant R., Dang-Nguyen T.Q., Noguchi J., Kaneko H., Kikuchi K. (2017). Effects of polyethylene glycol and a synthetic ice blocker during vitrification of immature porcine oocytes on survival and subsequent embryo development. Anim. Sci. J..

[139] Colombo M., Zahmel J., Jänsch S., Jewgenow K., Luvoni G.C. (2020). Inhibition of Apoptotic Pathways Improves DNA Integrity but Not Developmental Competence of Domestic Cat Immature Vitrified Oocytes. Front. Vet. Sci..

[140] Hwang I.S., Hara H., Chung H.J., Hirabayashi M., Hochi S. (2013). Rescue of vitrified-warmed bovine oocytes with rho-associated coiled-coil kinase inhibitor. Biol. Reprod..

[141] Li R., Murphy C.N., Spate L., Wax D., Isom C., Rieke A., Walters E.M., Samuel M., Prather R.S. (2009). Production of piglets after cryopreservation of embryos using a centrifugation-based method for delipation without micromanipulation. Biol. Reprod..

[142] Fu X.W., Wu G.Q., Li J.J., Hou Y.P., Zhou G.B., Lun S., Wang Y.P., Zhu S.E. (2011). Positive effects of Forskolin (stimulator of lipolysis) treatment on cryosurvival of in vitro matured porcine oocytes. Theriogenology.

[143] Jiang S., Zhang J., Jiang X., Tao Y., Yang J., Zhao K., Li N., Feng L., Shen H., Wang Y. (2025). Pre-incubation with levocarnitine alleviates vitrification and thawing damage in mouse oocytes. J. Assist. Reprod. Genet..

[144] Leal G.R., Oliveira T.A., de Paula Guimarães M.P., Correia L.F.L., Vasconcelos E.M., Souza-Fabjan J.M.G. (2024). Lipid modulation during IVM increases the metabolism and improves the cryosurvival of cat oocytes. Theriogenology.

[145] Raza S.H.A., Abd El-Aziz A.H., Abdelnour S.A., Easa A.A., Alagawany M., Farag M.R., Al-Mutary M.G., Elfadadny A., Khan R., Quan G. (2021). The role of forskolin as a lipolytic stimulator during in vitro oocyte maturation and the in vitro embryo production of livestock. Reprod. Domest. Anim..

[146] Oliveira C.S., Feuchard V., Marques S.C.S., Saraiva N.Z. (2021). Modulation of lipid metabolism through multiple pathways during oocyte maturation and embryo culture in bovine. Zygote.

[147] Ohata K., Ezoe K., Miki T., Kouraba S., Fujiwara N., Yabuuchi A., Kobayashi T., Kato K. (2021). Effects of fatty acid supplementation during vitrification and warming on the developmental competence of mouse, bovine and human oocytes and embryos. Reprod. BioMed. Online.

[148] Yu J., Li P. (2017). The size matters: Regulation of lipid storage by lipid droplet dynamics. Sci. China Life Sci..

[149] Houten S.M., Wanders R.J.A. (2010). A general introduction to the biochemistry of mitochondrial fatty acid β-oxidation. J. Inherit. Metab. Dis..

[150] Lane M., Maybach J.M., Gardner D.K. (2002). Addition of ascorbate during cryopreservation stimulates subsequent embryo development. Hum. Reprod..

[151] Zhang Z., Mu Y., Ding D., Zou W., Li X., Chen B., Leung P.C., Chang H.M., Zhu Q., Wang K. (2021). Melatonin improves the effect of cryopreservation on human oocytes by suppressing oxidative stress and maintaining the permeability of the oolemma. J. Pineal Res..

[152] Xiang D.C., Jia B.Y., Fu X.W., Guo J.X., Hong Q.H., Quan G.B., Wu G.Q. (2021). Role of astaxanthin as an efficient antioxidant on the in vitro maturation and vitrification of porcine oocytes. Theriogenology.

[153] Shirzeyli M.H., Eini F., Shirzeyli F.H., Majd S.A., Ghahremani M., Joupari M.D., Novin M.G. (2021). Assessment of Mitochondrial Function and Developmental Potential of Mouse Oocytes after Mitoquinone Supplementation during Vitrification. J. Am. Assoc. Lab. Anim. Sci..

[154] Xu H., Jia C., Cheng W., Zhang T., Tao R., Ma Y., Si L., Xu Y., Li J. (2020). The Effect of L-Carnitine Additive During In Vitro Maturation on the Vitrification of Pig Oocytes. Cell. Reprogramming.

[155] Gutierrez-Castillo E., Diaz F.A., Talbot S.A., Bondioli K.R. (2023). Effect of bovine oocyte vitrification with EGTA and post-warming recovery with resveratrol on meiotic spindle, mitochondrial function, reactive oxygen species, and developmental competence. Theriogenology.

[156] Wang C.L., Xu H.Y., Xie L., Lu Y.Q., Yang X.G., Lu S.S., Lu K.H. (2016). Stability of the cytoskeleton of matured buffalo oocytes pretreated with cytochalasin B prior to vitrification. Cryobiology.

[157] Moawad A.R., Zhu J., Choi I., Amarnath D., Campbell K.H. (2013). Effect of Cytochalasin B pretreatment on developmental potential of ovine oocytes vitrified at the germinal vesicle stage. Cryo Lett..

[158] Khodabandeh Z., Jamhiri I., Dehghani N., Daneshpazhouh H., Namavar Jahromi B., Dianatpour M., Alaee S. (2021). The effect of docetaxel on survival, fertilization rate and apoptosis-related genes mRNA expression in mouse metaphase II oocytes following vitrification. Iran. J. Vet. Res..

[159] Chasombat J., Nagai T., Parnpai R., Vongpralub T. (2015). Pretreatment of in vitro matured bovine oocytes with docetaxel before vitrification: Effects on cytoskeleton integrity and developmental ability after warming. Cryobiology.

[160] Zhou X., Li W., Zhang D., Dai J. (2015). Hydroxyapatite nanoparticles improved survival rate of vitrified porcine oocytes and its mechanism. Cryo Lett..

[161] Li W., Zhou X., Dai J., Zhang D., Liu B., Wang H., Xu L. (2013). Effect of hydroxyapatite nanoparticles on MII-stage porcine oocytes vitrification and the study of its mechanism. Sheng Wu Yi Xue Gong. Cheng Xue Za Zhi.

[162] Liu Q., Liu A., Liu Y., Li J., Bai J., Hai G., Wang J., Liu W., Wan P., Fu X. (2024). Hydroxyapatite nanoparticle improves ovine oocyte developmental capacity by alleviating oxidative stress in response to vitrification stimuli. Theriogenology.

[163] Srdjenovic B., Milic-Torres V., Grujic N., Stankov K., Djordjevic A., Vasovic V. (2010). Antioxidant properties of fullerenol C_60_(OH)_24_ in rat kidneys, testes, and lungs treated with doxorubicin. Toxicol. Mech. Methods.

[164] Chang X., Niu S., Shang M., Li J., Guo M., Zhang W., Sun Z., Li Y., Zhang R., Shen X. (2023). ROS-Drp1-mediated mitochondria fission contributes to hippocampal HT22 cell apoptosis induced by silver nanoparticles. Redox Biol..

[165] Wang L., McFadden J.W., Yang G., Zhu H., Lian H., Fu T., Sun Y., Gao T., Li M. (2021). Effect of melatonin on visceral fat deposition, lipid metabolism and hepatic lipo-metabolic gene expression in male rats. J. Anim. Physiol. Anim. Nutr..

[166] Lee S., Kim H.J., Cho H.B., Kim H.-R., Lee S., Park J.-I., Park K.-H. (2023). Melatonin loaded PLGA nanoparticles effectively ameliorate the in vitro maturation of deteriorated oocytes and the cryoprotective abilities during vitrification process. Biomater. Sci..

[167] Abbasi Y., Hajiaghalou S., Baniasadi F., Mahabadi V.P., Ghalamboran M.R., Fathi R. (2021). Fe3O4 magnetic nanoparticles improve the vitrification of mouse immature oocytes and modulate the pluripotent genes expression in derived pronuclear-stage embryos. Cryobiology.

[168] Zhan L., Hinnen H., Gopinathan K.A., Toner M. (2025). Autonomous cryoprotectant loading of the oocyte using microfluidic transistors. Device.

[169] Lin H., Liu C., Cao Y., Zhou X. (2025). Development of an automated device for the optimization of oocyte and embryo vitrification. Cryobiology.

[170] Qin J., Guo S., Yang J., Qazi I.H., Pan B., Lv T., Zang S., Fang Y., Zhou G. (2021). Melatonin Promotes in vitro Development of Vitrified-Warmed Mouse GV Oocytes, Potentially by Modulating Phosphorylation of Drp1. Front. Vet. Sci..

[171] Davoodian N., Kadivar A., Ahmadi E., Nazari H., Mehrban H. (2021). Quercetin effect on the efficiency of ovine oocyte vitrification at GV stage. Theriogenology.

[172] Edidin M. (2003). The state of lipid rafts: From model membranes to cells. Annu. Rev. Biophys. Biomol. Struct..

[173] Wozniak K., Reichelderfer R., Ghaemi S., Hupp D., Fuzesi P., Ringler G., Marrs R.P., Schiewe M.C. (2024). Ultra-fast vitrification and rapid elution of human oocytes: Part II—Verification of blastocyst development from mature oocytes. Reprod. BioMed. Online.

[174] Zeron Y., Sklan D., Arav A. (2002). Effect of polyunsaturated fatty acid supplementation on biophysical parameters and chilling sensitivity of ewe oocytes. Mol. Reprod. Dev..

[175] Cimadomo D., Cobo A., Galliano D., Fiorentino G., Marconetto A., Zuccotti M.P., Rienzi L.P. (2024). Oocyte vitrification for fertility preservation is an evolving practice requiring a new mindset: Societal, technical, clinical, and basic science-driven evolutions. Fertil. Steril..

[176] Cobo A., Diaz C. (2011). Clinical application of oocyte vitrification: A systematic review and meta-analysis of randomized controlled trials. Fertil. Steril..

[177] Cai H., Niringiyumukiza J.D., Li Y., Lai Q., Jia Y., Su P., Xiang W. (2018). Open versus closed vitrification system of human oocytes and embryos: A systematic review and meta-analysis of embryologic and clinical outcomes. Reprod. Biol. Endocrinol..

[178] Edgar D.H., Gook D.A. (2012). A critical appraisal of cryopreservation (slow cooling versus vitrification) of human oocytes and embryos. Hum. Reprod. Update.

